# Insights into the human mesenchymal stromal/stem cell identity through integrative transcriptomic profiling

**DOI:** 10.1186/s12864-016-3230-0

**Published:** 2016-11-21

**Authors:** Beatriz Roson-Burgo, Fermin Sanchez-Guijo, Consuelo Del Cañizo, Javier De Las Rivas

**Affiliations:** 1 Bioinformatics and Functional Genomics Group, Cancer Research Center (IBMCC, CSIC/USAL) and IBSAL, Consejo Superior de Investigaciones Cientificas (CSIC), Salamanca, Spain; 2 Hematology Department, IBSAL-Hospital Universitario de Salamanca, Salamanca, Spain; 3 Centro en Red de Medicina Regenerativa y Terapia Celular de Castilla y León, Salamanca, Spain

**Keywords:** Stromal cells, Mesenchymal stem cells, Placenta, Bone marrow, Adipose tissue, Human gene expression, Bioinformatic meta-analysis, Cytokines, CD marker

## Abstract

**Background:**

Mesenchymal Stromal/Stem Cells (MSCs), isolated under the criteria established by the ISCT, still have a poorly characterized phenotype that is difficult to distinguish from similar cell populations. Although the field of transcriptomics and functional genomics has quickly grown in the last decade, a deep comparative analysis of human MSCs expression profiles in a meaningful cellular context has not been yet performed. There is also a need to find a well-defined MSCs gene-signature because many recent biomedical studies show that key cellular interaction processes (i.e. inmuno-modulation, cellular cross-talk, cellular maintenance, differentiation, epithelial-mesenchymal transition) are dependent on the mesenchymal stem cells within the stromal niche.

**Results:**

In this work we define a core mesenchymal lineage signature of 489 genes based on a deep comparative analysis of multiple transcriptomic expression data series that comprise: (i) MSCs of different tissue origins; (ii) MSCs in different states of commitment; (iii) other related non-mesenchymal human cell types. The work integrates several public datasets, as well as *de-novo* produced microarray and RNA-Seq datasets. The results present tissue-specific signatures for adipose tissue, chorionic placenta, and bone marrow MSCs, as well as for dermal fibroblasts; providing a better definition of the relationship between fibroblasts and MSCs. Finally, novel CD marker patterns and cytokine-receptor profiles are unravelled, especially for BM-MSCs; with MCAM (CD146) revealed as a prevalent marker in this subtype of MSCs.

**Conclusions:**

The improved biomolecular characterization and the released genome-wide expression signatures of human MSCs provide a comprehensive new resource that can drive further functional studies and redesigned cell therapy applications.

**Electronic supplementary material:**

The online version of this article (doi:10.1186/s12864-016-3230-0) contains supplementary material, which is available to authorized users.

## Background

Stromal cells are connective tissue cells that support the functional part of an organ. The fibroblast (FIB) is the prime representative of a stromal cell type. Mesenchymal stem cells are adult, self-renewing multipotent progenitors that also inhabit the stromal compartment [1–4]. A population of stromal cells that demonstrate stem cell capabilities liable to be isolated from the bone marrow and from other diverse human tissues (like adipose, cartilage, muscle), is what we know as the Mesenchymal Stromal/Stem Cell population (MSCs) [5]. Specific traits that lead to the separation of uncommitted stages from differentiated ones are not yet conclusively deciphered. Nevertheless, the expanded settlement along the body of MSCs, the easiness of in vitro culturing, their differentiation capabilities, and their contained immuno-modulatory capacities have empowered them to be used in regenerative medicine for restoring the matrix or cellular elements of damaged tissues or for diminishing inflammatory or immune reactions. For these reasons, the MSCs have been also included in many recent cell therapy trials [6].

Cellular phenotypes can be defined by the expression and active contribution of specific genes. Therefore, many genome-wide profiling studies have been undertaken to answer unresolved questions over specific cell types. In the case of human MSCs, differentially expressed genes have been explored in pioneering studies by Wagner et al. [7] using global gene expression profiling. Different probe methodologies and discrepant experimental and analytic protocols interfere in the comparison between separate reports. However, as technology has been progressing, cell gene signatures have improved in sensitivity and specificity.

To characterise the specific identity features of MSCs, we took advantage of multiple accessible transcriptomic data gathered from different cell types with different degree of commitment. At the same time, we undertook a *de-novo* study based on new experimental data, generated to investigate the nature of MSCs and the inherent changes associated to their different tissue origins, variability that tissue-MSCs retain even during the first culture expansion stages [8, 9]. As a whole, the data collection produced to feed the performed study included 264 samples selected from public databases, a self-produced dataset of 15 samples analysed with high-density exon microarrays, and an additional set of six samples analysed with RNA deep-sequencing technology. The construction of a large transcriptomic framework of human stromal cells, together with their most related cell types, have facilitated to identify the relative differences and similarities between them.

Analysing the global gene expression profiles with a robust approach, we have been able to identify a polished signature comprising the common MSC lineage features in a set of 489 up-regulated genes. Functional linkage among signature genes also established the basal mesenchymal routines that cells normally trigger in their lifetime. Specific genes associated to each tissue were also scrutinised, specially the cytokine and the CD patterns. We have further explored the transcriptome of the bone marrow population of MSCs (BM-MSCs) and investigated the potential interactions with their niche-mates, the hematopoietic stem and progenitor cells (HSPCs). The exchanged signals and cross-talk interactions between these two, determines the establishment of the functional bone marrow microenvironment. Finally, by overlapping the results of our extensive data-driven exploration with other published signatures in a state-of-the-art compendium, we rescued genes that appear frequently reported, underlying the value of the MSCs characterisation presented.

## Results

### Cytological variations of stromal cells from different origins

**Fig. 1:**
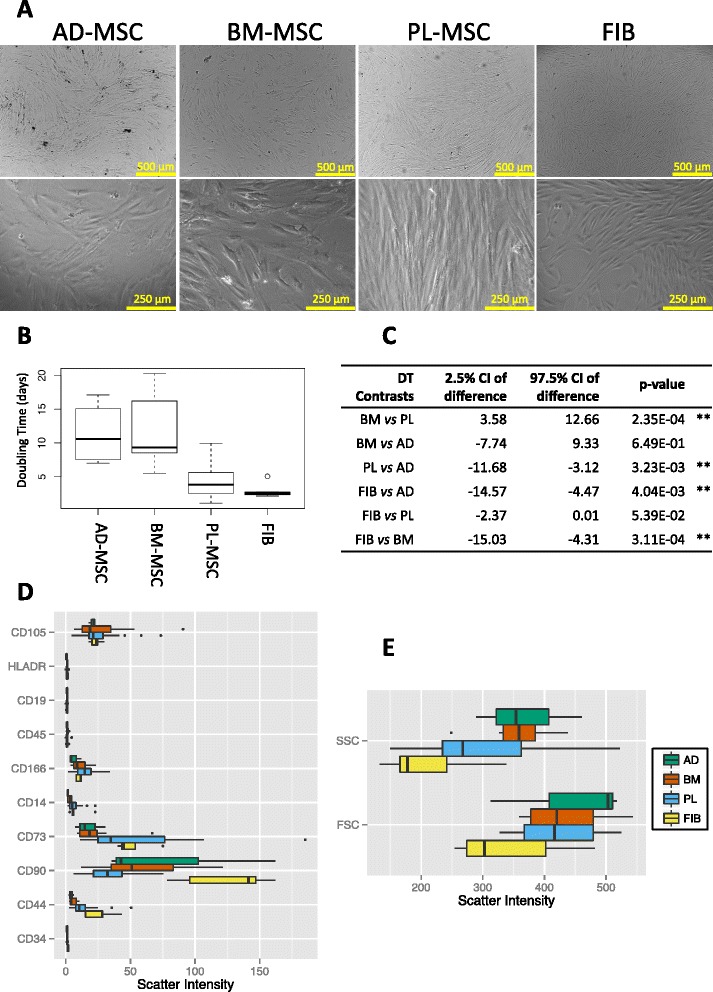
Characterization of MSCs following the ISCT criteria. **a** Microscope photographs of human stromal cells in culture taken at passage three: phase contrast micrographs seen at 4× and 10×. **b-c** Analysis of population doubling times: **b** boxplot of the doubling time distributions per stromal cell culture; **c** Wilcoxon test results of contrasted doubling times between stromal types. (DT = Doubling Time; W = Wilcoxon Test; CI = Confidence Interval). Labels for stromal cell types: AD = Adipose tissue MSC; BM = Bone marrow MSC; PL = Placental MSC; FIB = Dermal fibroblasts. **Significant p-values under 0.05. **d**-**e** Boxplots of normalized scatter intensities collected from flow-cytometry immunophenotyping assays using 10 different markers: CD105, HLADR, CD19, CD45, CD166, CD14, CD73, CD90, CD44 and CD34. (SSC = side scatter; FSC = forward scatter)

Primary cultures of stromal cells isolated from different origins included: MSCs from adipose tissue (AD-MSCs), MSCs from bone marrow (BM-MSCs) and MSCs from placental tissue (PL-MSCs); as well as fibroblasts (FIB) from dermal tissue. Under the microscope, the fibroblastic spindle-shape of stromal cells appeared clearly manifest along the study cultures (Fig. 1a). Some peculiarities in cell morphology may certainly be appreciated between stromal cells from different tissue origins. The placental MSCs were the longest, similar to fibroblasts. BM and AD-MSCs were difficult to distinguish and had a more irregular morphology in culture, with cells that mixed fusiform shapes with less elongated star shapes.

In terms of cell growth rates, population-doubling times were significantly shorter on fibroblasts, followed by PL-MSCs and later AD- and BM-MSCs. Between these last two, no significant differences were found. Wilcoxon test p-values point out the differences in growth rate between cultures (see boxplot and table in Fig. 1b, c). The complete data collected from cultures is available in Additional file 1 *.*

The intensity of standard CD markers detected by flow cytometry assays in different stromal cells is presented in comparative boxplots in Fig. 1d. CD90 showed the strongest signal and, strikingly, the highest in fibroblasts and the lowest in PL-MSCs among the cellular classes. CD90 together with CD44 resulted the most discriminating for FIBs from the rest of the MSC populations. Of less variability resulted CD73, been similar to AD- and BM-MSCs, and expressed stronger in PL-MSCs and FIBs. CD105 showed similar expression along all cell populations. Forward scatter and side scatter measures also denoted differences (Fig. 1e). These cytometric parameters showed that fibroblasts are the smallest and less internally complex cells.

The complete panel of cytological assays defined by the ISCT confirmed the characteristic phenotype of mesenchymal stromal cells. Histograms of cytometry assays for each sample type are supplied in Additional file 2 *.* Confirmatory microscopic snapshots of tri-lineage differentiations of the three tissue-MSCs are also supplied in Additional file 3. To address the transcriptomic study of all these cell types, the produced and verified primary cultures of stromal cells were used to generate an *in-house de-novo* gene expression dataset.

### Meta-analysis of multiple sample series of MSCs and other related cell types

To begin the identification of human mesenchymal stromal cells, we first searched in multiple public databases to find a collection of datasets that could be assembled and analysed in a common framework. In this way, we integrated several transcriptomic data series and carried out a meta-analysis study on them. We first explored the genome-wide expression profiles to better visualise the relationships of MSCs to differently related cell types: from same lineage derived osteoblasts, adipoblasts, chondroblast or fibroblasts, to lineage flipped hematopoietic progenitors (HSPCs) and differentiated cells (e.g. lymphocytes), and cells from foetal origin.

The meta-analysis study approach allows increasing the number of samples available, thus improving the robustness of the results. In our case, a meta-set of 264 expression microarrays from 18 independent data series were selected through a discriminating process that included a total initial collection of more than 500 samples (Additional file 4). A benchmark of several biological backgrounds was created, including samples from the mesenchymal lineage, together with samples from the hematopoietic lineage. Cells from the hematopoietic system presented a well-characterised immunophenotype that defined each cellular population along the maturation process. This brought a well-known system that served as distant control group to compare the less phenotypically characterised cells from the stromal lineage. Differentiated cells of both lineages, even from unrelated tissues, were included whenever available, to provide a broader profiling.

**Fig. 2:**
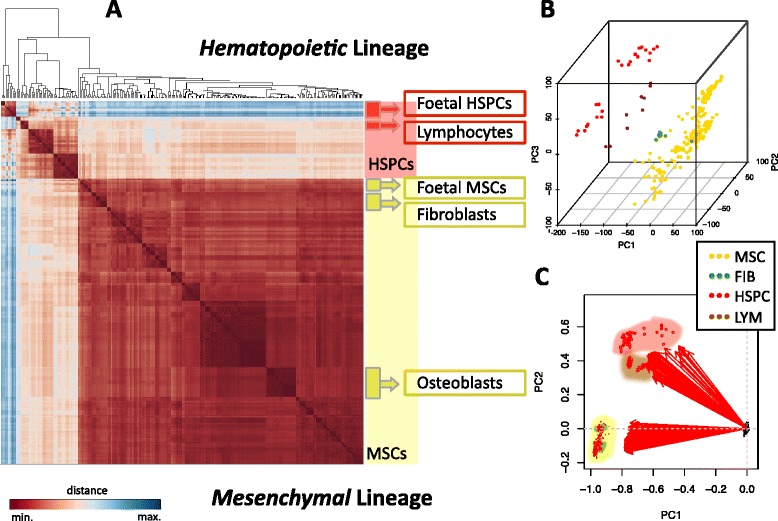
Meta-analysis of MSCs and related cell types. **a** Sample-to-sample heatmap of 264 microarrays that includes mesenchymal and hematopoietic lineage cells. Clustering of samples relies upon Euclidean distances derived from the pairwise calculation of the *Pearson* correlation between the expression vectors of each sample. In the right panel: the hematopoietic lineage samples are shaded in *red* and mesenchymal lineage samples are in *yellow*. The colour scale represents shorter to larger distance: from *dark red* to *dark blue*. **b** 3D plot of PCA based on the gene expression of the stromal and hematopoietic cells studied. Each dot represents a sample microarray from the repertoire of 264 and includes the global gene expression for each sample. **c** *Biplot* of PCA performed by genes of the same data. Samples are represented as arrow vectors in the derived PCA space on the *Biplot*

The transcriptomic profiles produced provide a view of the relationships between cells, considering differences and similarities as distances or proximities that the cells exhibit, associated to the functional backgrounds they come from. A non-supervised hierarchical clustering analysis of global expression, based on *Pearson* correlations, is represented in the heatmap in Fig. 2a. The heatmap shows that hematopoietic (marked in red) and mesenchymal (marked in yellow) lineages fall clearly separated in two main clusters. Samples from foetal origin, included inside both lineages, are segregated from their other relatives. In this way, the distribution of samples is primarily associated to the cellular lineage, as well as their differentiation state and tissue of origin. A very similar biologically distributed clustering is preserved when performing a principal component analysis (PCA). Information contained in the principal components is exposed in Fig. 2b and c. A 3D plot in Fig. 2b displays the distribution of 264 samples within the space of the three first components of the PCA using genes as variables. The cumulative variance explained by these three components is 33.28%, 45.75% and 56.11%. A meaningful reading of these PCA results is the agreement between the unsupervised clusterisation –based on the whole genome expression– and the biological cell types studied. The main variance accumulated in the first PC primarily explains the cellular lineage variation. The second and third PCs further explain the cell type specificity intrinsic to each lineage. Using samples as variables, another PCA was done and the cumulative variance explained by the first components was: PC1 81.72%, PC1 + PC2 88.11% and PC1 + PC2 + PC3 90.21%. The *Biplot* presented in Fig. 2c shows the two principal components representing 88% of the variability. Thus, PC1 and PC2 account for the main separation between the two types of cellular lineages: stromal cells in one part and hematopoietic cells in the other. The coherence with biology of sample distributions also manifests that batch-effects are not relevant in the data. Thus, the pre-processing and normalisation steps have been well accomplished, making the collection suited for upcoming differential expression comparisons and further integrative analysis (presented in the following sections of the Results).

Clustering and PCA analyses performed over the global expression profiles show that the transcriptomes appear associated by their biological context of origin, with a clear segregation between the hematopoietic cell lineage and the mesenchymal cell lineage, as well as clear proximity of the different cell types assigned to the mesenchymal lineage: adipoblasts, chondroblasts, fibroblasts and osteoblasts. An interesting result is the way differentiated cells cluster with respect to the progenitor cells. Lymphocytes get a position separated from their hematopoietic progenitors. However, fibroblasts do not appear very much segregated from other stromal cells in this comparison, and mesenchymal samples gather closer than the samples from hematopoietic cells. This proximity occurs even considering that most of the MSCs included here come from the bone marrow; however fibroblasts come from the dermis, two distant tissues that do not seem to mark as important differences in terms of whole gene expression as the distance observed between the stromal and the hematopoietic progenies.

With respect to the samples of foetal origin, in the case of HSPCs there is a clear difference between the foetal and adult origin, but in the case of MSCs the expression profiles are much less different (Fig. 2a). The larger difference observed in the case of HSPCs should be expected, since it is known that human foetuses have a different hematopoiesis to adults because prenatal hematopoietic stem cells are formed in multiple anatomical sites (faetal liver, placenta, etc.), and they only colonise the bone marrow at birth to establish a normal hematopoiesis during postnatal life. In this way also, the lymphocytes and the immune system are still not fully developed in the embryonic stages and foetal erythrocytes express several specific proteins, like “foetal haemoglobin”, that are not present in adult blood.

### Transcriptomic homology between MSCs from different tissue origins, FIB and HSPCs

Since the first descriptions of MSCs in the bone marrow [1, 10], many tissues have been disclosed as sources of MSCs: muscle, skin, adipose tissue, umbilical cord blood, *Wharton’s* jelly, placenta, etc. We focused the following analyses to the characteristics that the different tissue basements confer to MSCs. We produced an *in-house* genome-wide expression dataset with MSCs from three different tissue origins. The platform *Affymetrix Human Exon 1.0* was used since it allowed the expansion of the range of gene loci assayed from ~17 k (in older *Affymetrix* platforms included in the meta-analysis) to ~20 k genes.

Cultured populations of MSCs from adipose tissue (AD), bone marrow (BM) and placenta (PL) were submitted to genome-wide expression profiling, together with skin fibroblasts (FIB) and HSPCs as contrast cell types. As shown in the preceding results, fibroblasts present a close relationship to the populations of MSCs and the rest of the stromal lineage cells. On the other hand, HSPCs provide a different lineage contrast related to stem cells, but not to the mesenchymal lineage.

**Fig. 3:**
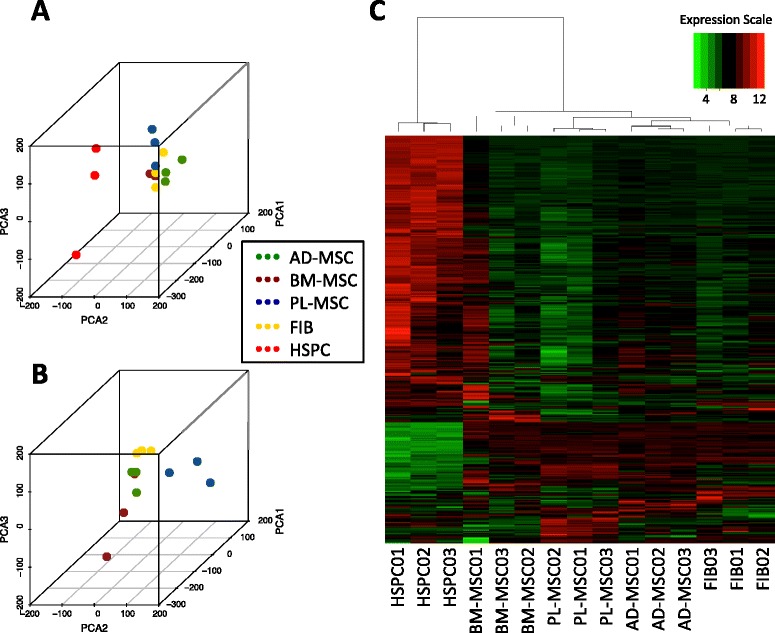
Analysis of tissue-MSCs in a *de-novo* dataset of 15 microarrays. **a** 3D plot of PCA performed for samples of all the cells under study (15 arrays) using the whole gene expression profiles. **b** 3D plot of PCA performed only with the stromal cell samples (12 arrays) removing the hematopoietic cells. **c** Heatmap of semi-supervised clustering based on the 358 CD marker genes. (The expression scale is in log2-transformed intensity values. The microarrays platform used was:*Affymetrix Human Exon 1.0*)

Principal component analysis of the 15 samples (Fig. 3a) reproduced the behaviour observed in the meta-analysis: clear segregation of HSPCs from the mesenchymal lineage, and close association of fibroblasts to the rest of the MSCs from the three different tissues. The cumulative variance explained by the 3 components here represented was: 33.61, 50.76 and 58.62%. In addition, another PCA including only MSCs and fibroblasts reveals a clearer separation of the cell types, showing the fibroblasts in a most-compacted cluster with less sample-to-sample variability than the rest of the MSCs (Fig. 3b). The cumulative variance explained in this case was: PC1 33.93%, PC1 + PC2 46.12% and PC1 + PC2 + PC3 56.27%.

To observe the system through a narrower window, we performed a semi-supervised clustering analysis considering only the collection of 358 CD genes, defined as clusters of differentiation targetable for cell immunophenotyping (Fig. 3c). These known cell surface marker proteins allow the display of cell relationships focusing on the currently available phenotypical knowledge. The corresponding dendrogram in the upper margin of the heatmap (Fig. 3c) shows that the cellular entities were well segregated, the replicates were not mixed with each other, whilst the three subtypes of tissue-MSCs were closely clustered. Interestingly, BM-MSCs and not fibroblasts are the first population more separated within the stromal repertoire when CD markers are used for sample clustering. Analysis of the samples clustering based on all-genes (i.e. unsupervised clustering of samples based on *Pearson* correlations of the expression along all genes) indicates that BM-MSCs are within the tissue-MSCs cluster (Additional file 5). These broad clustering analyses based on the whole genome-wide expression profiles show only the main trends, but deeper analysis of the specific genes activated in each cell type and subtype should be performed to achieve better definition of stromal cells phenotype and understand their similarities to other cells.

### Differential expression (DE) signatures that define tissue specificity of MSCs

Going further within the transcriptomic profiling and as a way to evaluate the similarity between MSCs, we tackle the idea of establishing the number of differentially expressed (DE) genes as a measurable value of cell population distances. First, we present the results derived from the meta-analysis of 119 samples obtained from 16 different published datasets, that included a collection of 50 BM-MSC samples (Additional file 4). These analyses were done using a re-sampling method (as indicated in Methods) that allows finding the most stable differential expression gene signatures. Three main contrasts were designed to compare BM-MSCs against other cell types: (1) BM-MSCs versus HSPCs, (2) BM-MSCs versus skin Fibroblasts, and (3) BM-MSCs versus Osteoblasts. A summary describing all these comparisons and the DE outcomes is included in Additional file 6. This approach yielded a value of 401 DE genes between BM-MSCs and FIB, with 207 up-regulated and 194 down-regulated in BM-MSC. The contrast between BM-MSCs and HSPCs yielded 4748 DE genes (2124 up-regulated and 2624 and down-regulated, in BM-MSC). As expected, broadly used MSC markers CD73 (NT5E), CD90 (THY1) and CD105 (ENG, Endoglin) were found up-regulated along the listed genes. Table 1 presents 28 CD marker genes found in the complete list of significant genes. When we crossed lists of up-regulated genes in BM-MSCs from both contrasts (versus FIBs and versus HSPCs), one CD marker gene (i.e. CD146) and two transcription factors (i.e. ID3 and PAWR) remained in the intersection. The MCAM glycoprotein gene (CD146) has been postulated as a marker of MSCs population in the sub-endothelial niche within the bone marrow [11, 12].

**Table 1:** CD marker signature defined for BM-MSCs

CD identifier	Gene aliases	Description
**CD13**	**ANPEP; PEPN**	**Aminopeptidase N (Microsomal aminopeptidase) (Gp150)**
CD49c	ITGA3	Integrin alpha-3 (Integrin VLA-3 alpha subunit) (Galactoprotein b3)
**CD49e**	**ITGA5; FNRA**	**Integrin alpha-5 (Integrin VLA-5 alpha subunit) (Fibronectin receptor alpha subunit)**
**CD58**	**CD58; LFA3**	**Lymphocyte function-associated antigen 3 (Surface glycoprotein LFA-3)**
CD63	CD63; MLA1	CD63 antigen (Melanoma-associated antigen ME491) (LAMP-3) (Ocular melanoma-associated antigen) (OMA81H) (Granulophysin) (Tetraspanin-30)
**CD73**	**NT5E; NT5; NTE**	**5′-nucleotidase**
**CD90**	**THY1**	**Thy-1 membrane glycoprotein**
**CD105**	**ENG; END**	**Endoglin**
CD107a	LAMP1	Lysosome-associated membrane glycoprotein 1 (LAMP-1)
CD107b	LAMP2	Lysosome-associated membrane glycoprotein 1 (LAMP-2)
**CD140b**	**PDGFRB**	**Beta-type platelet-derived growth factor receptor (PDGF-R-beta)**
**CD146**	**MCAM; MUC18; CD146**	**Cell surface glycoprotein MUC18 (Melanoma cell adhesion molecule) (Melanoma-associated antigen A32) (S-endo 1 endothelial-associated antigen) (Cell surface glycoprotein P1H12)**
CD147	BSG	Basigin (Leukocyte activation antigen M6) (Collagenase stimulatory factor)
CD156c	ADAM10; MADM	ADAM 10 (A disintegrin and metalloproteinase domain 10)
CD164	CD164	Putative mucin core protein 24 (Multi-glycosylated core protein 24)
CD167b	DDR2; NTRKR3	Discoidin domain-containing receptor 2 (Tyrosine-protein kinase TYRO 10) (Neurotrophic tyrosine kinase, receptor-related 3)
CD213a1	IL13RA1; IL13RA	Interleukin-13 receptor alpha-1 chain
CD222	IGF2R; MPRI	Cation-independent mannose-6-phosphate receptor (Insulin-like growth factor II receptor)
CD230	PRNP	Major prion protein (PrP) (PrP27-30) (PrP33-35C) (ASCR)
CD248	CD248; TEM1	Endosialin (Tumour endothelial marker 1)
CD266	TNFRSF12A; FN14	Tumour necrosis factor receptor superfamily member 12A (Fibroblast growth factor-inducible immediate-early response protein 14) (Tweak-receptor)
CD280	MRC2; ENDO180	Macrophage mannose receptor 2 (Urokinase receptor-associated protein) (Endocytic receptor 180)
CD284	TLR4	Toll-like receptor 4
CD292	BMPR1A; ACVRLK3	Bone morphogenetic protein receptor type IA (Serine/threonine-protein kinase receptor R5) (SKR5) (Activin receptor-like kinase 3) (ALK-3)
CD304	NRP1; NRP	Neuropilin-1 (Vascular endothelial cell growth factor 165 receptor)
CD317	BST2	Bone marrow stromal antigen 2 (BST-2)
CD331	FGFR1; FGFBR	Basic fibroblast growth factor receptor 1 (bFGF-R) (Fms-like tyrosine kinase 2)
CDw210b	IL10RB; CRFB4	Interleukin-10 receptor beta chain (IL-10R2) (Cytokine receptor class-II member 4)

Bold font indicates CDs previously reported or used as markers for BM-MSCs (update taken from Calloni’s et al. review [21])

To better prove the value of the differential expression signatures obtained, we used again the re-sampling tool to perform exploratory tests upon some training samples of the microarray collection. Unrelated mesenchymal samples following osteogenic induction, either in vivo or using certain effector molecules, were submitted to the re-sampling process (see Additional file 6 contrasts 3, 4A, 4B and 4C; and plots in Additional file 7). These re-sampling analyses yielded inconsistent numbers of differentially expressed genes between conditions, with no significant outcome in some cases (e.g. contrast 4C). Such anomalous results could be due to the comparison of very similar cell types –that would not have any significant gene change– or the comparison of very heterogeneous and disperse cellular states. The heterogeneity could come from stochastic responses to the different stimulus that were applied to the stromal cells studied (see, for example, differential expression re-sampling curves in contrast 4A: BM-MSC versus dOST; and in contrast 4C: OSTB versus stOSTB) (Additional file 7). These results prove the high sensitivity of the method to sample alterations, and, thus, the robustness to find the most stable genes in each differential expression contrast. This stability also adds value to the signatures obtained by re-sampling, i.e. the ones produced in the first and second contrasts (contrasts 1 and 2, Additional file 7).

An enrichment analysis directed to explore the functional and biological meaning of the differentially expressed genes in MSCs is included in Table 2. When compared to HSPCs, certain functions are enriched in the BM-MSCs gene signature: *blood vessel development, vesicle localisation, cell migration, cell death regulation*. Moreover, *hematopoiesis* and *leukocyte activation* processes are enriched in HSPC genes. Significantly, in the comparison with fibroblasts, more BM-MSC genes appear annotated to bone development tasks (e.g. *skeletal system development*) and skeletal fibre organisation (e.g. *Z disc* and *myofibril*). By contrast, fibroblasts showed a clear enrichment in genes related to the organisation and function of the *extracellular matrix*.

**Table 2:** Functional enrichment analysis of DE genes obtained from the meta-analysis approach

Annotation term	Hits (gene symbols)	Hits	Term hits (%)	Adj.p-val (Benjamini)
Enriched functions in 2124 genes overexpresed in BM-MSCs compared to HSPCs				
cytoplasmic membrane-bounded vesicle	SEPT5, TGOLN2, SEC31A, FSTL3, ITSN1, C14ORF1, APP, BDNF, DAB2, PICALM, SCAMP1, BSG, MYO6, ACTN1, OPTN, SSPN, SPAG9, VEGFC, SERPINF1, RAB14, TRAPPC4, PDCD6IP, COPZ2, CAV2, RAB7A, PAM, COPZ1, ATP6V1B2, ITGB1, CALU, TIMP1, STX12, SLC30A5, TMED10, FN1, AP2M1, PLAT, P4HB, BECN1, GARS, WIPI1, DVL1, LAMP1, LAMP2, PPIB, YIPF3, YIPF5, HSP90AB1, COPA, CLTA, CLTB, SEC24A, RAB5B, AP2S1, HEXB, AP3S1, GJA1, PDIA6, PDIA4, CLTC, CANX, RABAC1, SLC1A5, COPB2, AP2B1, TPP1, MAPKAP1, GOPC, GOLGA5, ATP6V0D1, SEC24D, STX6, SEC23A, ADAM10, STX2, PIK3C2A, STXBP1, TMEM187, BGN, IGF2R, ARCN1, RAB5A, SORT1, CTSD, CTSB, COPG, COPE, GANAB, ANXA6, CD9, TMED2, CRISPLD2, TMEM33, CAMK2D, RAB11A, SNAP23, GPNMB, THBS1, EHD1, THBS2, EHD3, HSPA8, THBS3, PHLDA1, YWHAB, SPARC, TMEM168, ANXA2, NCSTN, SH3BP4, SCFD1, HSP90B1, LRP1, SMPD1	114	5.73	8.84E-07
establishment of vesicle localization	SEPT5, BBS4, COPA, MAP2K1, BBS7, COPZ1, WIPI1, COPB2, ARF1, PSEN1, ARCN1, TMED10, SNAP23, COPG, YKT6, COPE	16	0.80	1.72E-04
blood vessel development	RTN4, NRP1, HTATIP2, PGF, PRRX1, ANPEP, MMP2, CXCL12, CITED2, MAP3K7, AKT1, SHB, ATG5, CTGF, ANG, ROBO1, SEMA3C, RHOB, LOX, NR2F2, FGF2, CYR61, RECK, MYH9, SLIT2, THY1, VEGFC, BGN, HIF1A, NUS1, PSEN1, COL1A2, FOXC1, COL1A1, ACVR1, CAV1, TNFRSF12A, COL3A1, CDH2, TCF7L2, SEMA5A, PTK2, ITGAV, CHM, THBS1, PPAP2B, C1GALT1, RASA1, PLAT, EPAS1, MYO1E, TGFBR2, COL5A1, ANXA2, CDH13, GPI, LAMA4, PKNOX1, ENG, PLAU	60	3.02	5.06E-06
apoptosis	DLC1, TSPO, HRAS, MEF2A, SGMS1, ITSN1, MAGED1, SHB, CLPTM1L, APP, CDKN2A, RAD21, ATG5, UNC5B, GATA6, FAS, DAP, CUL1, CIB1, TWIST1, FADD, LIG4, BCAP31, PDCD6IP, NEK6, BLCAP, TNFRSF12A, RRAGA, BCL2L2, STK17A, RRAGC, PEA15, PRUNE2, DOCK1, TCTN3, ABR, TM2D1, LGALS1, SRA1, TRIO, FXR1, VDAC1, NCKAP1, BFAR, VCP, RABEP1, RTN4, HTATIP2, SGPP1, ZMAT3, BNIP3, GJA1, PAWR, DAXX, RTN3, AKT1, TNFRSF11B, DYNLL1, PAK2, BOK, GSN, BAG3, BAG2, RHOB, FGF2, MYC, MAGEH1, NDUFS1, DHCR24, LTBR, NOL3, ARHGEF12, CDK5, ECT2, ELMO2, ZDHHC16, EYA2, PSEN1, AVEN, PSME3, UBE2Z, ITM2B, GLRX2, TNFRSF1A, BCAP29, THBS1, PHLDA3, PHLDA2, ERCC2, PHLDA1, ACTC1, IL6, DNM1L, TMBIM6, UBE4B, YWHAB, BAD, STAT1, PLEKHF1, NCSTN, BNIP3L, SULF1, PERP	103	5.18	2.75E-03
cell migration	CTHRC1, JUB, NRP1, CXCL12, CTGF, ANG, ROBO1, SEMA3C, CAP1, NR2F2, FGF2, TWIST1, PRKCA, PTPRK, SATB2, ARID5B, EMX2, NRD1, MYH9, CDK5, SLIT2, VEGFC, HIF1A, TNS1, PSEN1, CFL1, SIX1, PDGFRB, FOXC1, LAMC1, ACVR1, CAV2, CCL2, NDN, FUT8, TNFRSF12A, ITGA11, KITLG, CDH2, ITGB1, VCAM1, PTK2, PAFAH1B1, PPAP2A, THBS1, PPAP2B, APC, FN1, PLAT, IL6, MET, COL5A1, CDH13, FYN, ITGA5, LRP6, APBB2, ENG, PLAU, MYH10	60	3.02	2.36E-04
Enriched functions in 2624 genes overexpressed in HSPCs compared to BM-MSCs				
regulation of leukocyte activation	LST1, HMGB3, BLM, STAT5A, IL18, SNCA, SPINK5, SART1, CD74, ADA, IL31RA, IL1B, MS4A2, IL2RG, INPP5D, HLA-DOA, TRAF6, LAG3, CD28, SYK, FCER1A, PTPRC, IL2RA, GIMAP5, IKZF1, FLT3, SLA2, CTLA4, STXBP2, IDO1, NFAM1, PRKCQ, CD38, CD83, CORO1A, CD86, TNFSF13B, LAX1, PRAM1, RIPK2, IRF4, VSIG4, SASH3	43	1.92	9.33E-04
hemopoiesis	LMO2, STAT5A, JAG2, TPD52, IL31RA, CDC42, SYK, RHOH, MB, EGR1, TTC7A, LYN, EOMES, NFAM1, DHRS2, CD40LG, AICDA, ADD2, CALCR, GPR183, BLM, KIT, ZBTB16, SOX6, TRIM10, CD74, ADA, TAL1, DOCK2, CHD7, RASGRP4, BCL2, BCL11A, TRAF6, CD28, PTPRC, GIMAP5, IKZF1, PLEK, EPB42, FLT3, HCLS1, HDAC5, HOXB4, RPL22, PLCG2, IRF8, IRF1, IRF4, SPTA1, CD79A, KLF1	52	2.33	6.28E-03
positive regulation of T cell activation	PTPRC, IL2RA, GIMAP5, IKZF1, BLM, STAT5A, IL18, ADA, CD74, SART1, CD83, PRKCQ, CORO1A, CD86, TNFSF13B, RIPK2, IL1B, IL2RG, TRAF6, SASH3, CD28, SYK	22	0.98	2.44E-02
Enriched functions in 194 genes overexpressed in skin fibroblasts compared to BM-MSCs				
extracellular matrix	EGFL6, LGALS1, MMP27, GRIA3, EMILIN2, MMP3, NTN1, MMP1, WNT2, FBLN2, FBLN5, F3, FBN2, MFAP4	14	7.57	5.22E-03
calcium ion binding	S100A4, F10, MASP1, LDLR, EGFL6, SCUBE2, MMP27, COLEC12, GALNTL1, MMP3, SLIT2, MMP1, DCHS1, STAT2, NPTX1, FBLN2, SVIL, FBLN5, ANXA11, CCBE1, FBN2, MFAP4, GALNT14	23	12.43	2.06E-02
EGF-like, type3	F10, LDLR, EGFL6, SCUBE2, FBLN2, FBLN5, CCBE1, ADAM33, FBN2, SLIT2	10	5.41	1.44E-02
Enriched functions in 207 genes overexpressed in BM-MSCs compared to skin fibroblasts				
system development	INHBA, RBP4, CTGF, DLX5, CHST11, FHL2, SORT1, MGP, FOXC1, NPR3, COL5A2, ANKH	12	6.32	5.08E-01
Z disc	SORBS2, PDLIM5, DMD, FHL2, HOMER1	5	2.63	2.77E-01
myofibril	SORBS2, PDLIM5, DMD, FHL2, HOMER1, TPM1	6	3.16	2.24E-01

Annotation Term: name and identifier of the annotated term in a functional database. Hits: (Observed Hits) number of genes from the DE gene list in each specific annotation term. Adj.p-val: (Adjusted *p*-values) *p*-values from functional enrichment analysis were adjusted using the Benjamini and Hochberg method. Gene symbols: identifiers of genes asigned to each functional term. Analyses were performed using DAVID web tool

**Fig. 4:**
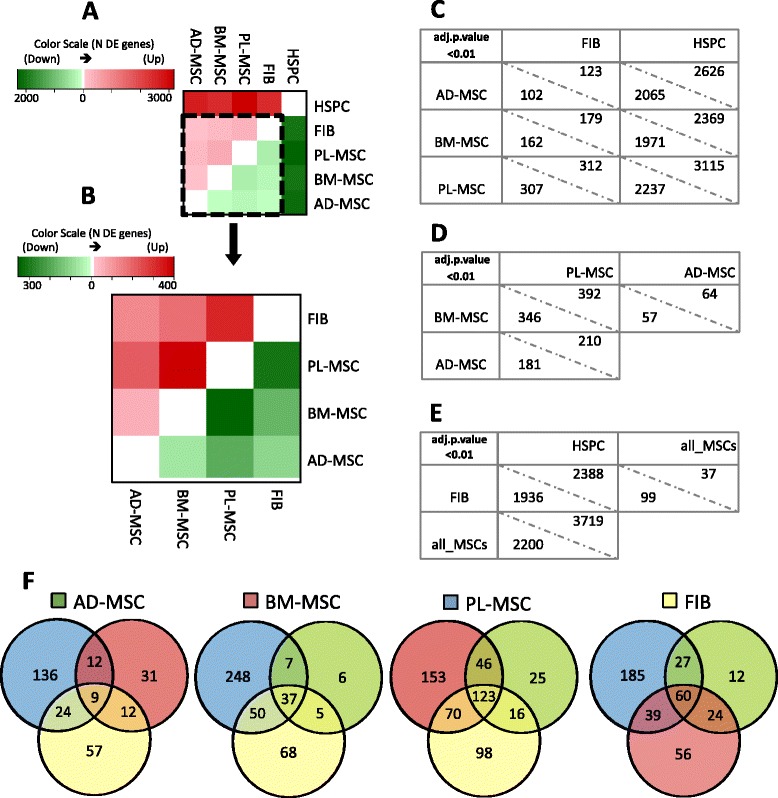
Differential expression analysis from the *de-novo* study of 15 microarrays. **a** Heatmap of distances measured as the number of differentially expressed genes between each cell type. *Red* scale accounts for up-regulated genes in the cell type labelled in the right panel; whilst *green* intensities represent down-regulated genes. The more intense the colour, the greater the distance between cell types involved. **b** Same as **a**, but HSPCs were removed to improve the visualisation of the differences between stromal cell types. **c-d-e** Tables presenting the number of differentially expressed genes per pair-wise comparison, indicating the up-regulated genes in each type (e.g. in **d**, BM-MSC versus PL-MSC: 346 genes UP in BM and 392 in PL). **f** *Venn* diagrams showing the number of genes that are up-regulated in each contrast against: AD-MSC (*green*), BM-MSC (*red*), PL-MSC (*blue*) and FIB (*yellow*)

Differential expression (DE) analyses were also performed in our *in-house* controlled dataset, which includes MSCs from three different sources. Figure 4a and b show two red-green heatmaps where the intensity increases proportionally to the number of DE genes between each contrast performed. The upper panel (Fig. 4a) includes the HSPCs, that are removed in the lower panel (Fig. 4b) to strengthen the comparisons between stromal cell types. In this comparison, the strongest differences were observed between PL-MSCs against all other stromal cells. This result may reflect the foetal nature of the placental cells. The opposite occurs for AD-MSCs, which show the lowest number of DE genes. This may indicate that this cell subtype in the adipose niche needs to prompt fewer specific genes to perform their tissue-specialised tasks.

The exact number of DE genes for all the pair-wise comparisons are presented in three tables in Fig. 4c, d, e. An additional contrast class, formed by the three tissue MSC samples, is also included in these analyses. This ‘all-MSCs’ class shows 5,919 DE genes against HSPC, but only 136 DE genes against FIB. These numbers may be considered a distance measurement between the cellular entities studied. Such values are similar and proportional to the measures obtained in the meta-analysis of public datasets, strengthening the reliability of our biological observations.

The described results bring forward the question of which genes might be specifically associated to each tissue origin of the MSCs analysed. To withdraw the potential tissue specific genes, we extracted the common DE genes of each MSC class across different comparisons. Intersections of DE gene lists are presented in Venn diagrams in Fig. 4f, where we only include the genes that were up-regulated in the comparisons of the indicated cell type with the others, for example for AD-MSCs: 181 genes versus PL-MSCs, 64 genes versus PL-MSCs and 102 genes versus FIB. From all these intersections we observed that, proportional to DE values, PL-MSCs present the largest list of tissue specific genes and AD-MSCs the shortest: 123 and 9, respectively, differentially expressed in all the comparisons against the rest of stromal cells. These genes encountered in the intersections will be very specific to each tissue-MSC. The lists corresponding to these genes are provided in Table 3. Some genes found are: VCAM1 (CD106) in BM-MSCs; NCAM1 (CD56) and DNAM1 (CD226) in PL-MSCs; PPAPDC1A in AD-MSCs (i.e. phosphatidate phosphatase involved in fatty acid metabolism); TWIST1 and TWIST2 in FIB (known as mesodermal determinant factors that block bone specification fate) [13].

**Table 3:** Tissue specific gene signatures: list of genes appearing differentially expressed in each tissue-MSC and fibroblasts when contrasted to each other

Tissue-stromal cells	Number of genes	Specific genes
AD-MSC	9	AC104654.2, AP000695.2, AP000843.1, MATN3, MFAP3L, PCDH9, **PDLIM3**, PPAPDC1A, Z69713.1
BM-MSC	37	ACAN, **AGT,** ANK3, AP001422.1, C20orf103, C20orf197, C5orf23, CHRDL1, **CXCL16**, DLX5, ENTPD1, EPHX1, **EYA1, EYA2, EYA4**, FBXO16, FMO3, HEY2, IGF2, IGFBP2, ITGA7, JAG1, KCNMB1, KIAA1217, KLHL3, LBP, **LEPR,** MAOB, **NOTCH3,** NPR3, RP11-145A3.3, SFTA1P, SLPI, TM4SF20, **VCAM1 (CD106)**, ZNF423
PL-MSC	123	AC003092.2, AC012409.1, AC026250.2, AC069155.1, AC090625.2, ADAM23, ADRA1D, AL121578.3, ALDH1A1, ALDH1A2, AMIGO2, ANO4, AR, ATRNL1, BRIP1, C10orf57, C12orf59, C16orf52, C3orf72, C9orf167, CACNA1H, CADPS2, CAMK1G, CAPN6, CARD16, CKS2, CNTN4, CTSH, CYTSB, DGKH, **DNAM1 (CD226)**, DTNA, E2F7, ETV4, F2RL1, FABP4, FAM105A, FAM155A, FAM60A, FJX1, GAS2L3, GBP4, GCLC, GLRXP3, GPR126, GPR37, HMGA2, HOXD10, HSD17B2, HTR1D, HTR2B, IFIT2, IFIT3, INA, KCNA3, KRT19, LAMA1, LCP1, LIMS1, LPCAT2, LRRC1, MAP3K5, MAPK8, MCTP1, MSI2, MUM1L1, **NCAM1 (CD56),** NEFM, NETO2, NID1, NNAT, NR2F1, PAMR1, PCDH10, PDCD1LG2, **PDGFC**, PLAT, PLBD1, PLCXD3, PMAIP1, RARB, RASGRP1, REN, RP11-117P22.1, RP11-251 J8.1, RP11-332P22.1, RP11-548O1.1, RP11-87H9.2, RP4-706A16.2, RPS6KA6, S1PR3, SCN9A, SERPINB2, SGIP1, SLC16A4, SLC1A2, SLC4A8, SLITRK4, SNCA, SNCAIP, SNORD27, SOCS1, SORBS1, SOX5, SSTR1, ST6GALNAC5, STRA6, SYPL2, SYT14, SYT16, SYTL5, TFPI2, TMEM154, TMEM51, **TNFSF15, TNFSF18, TNFSF4,** TRHDE, TRPC4, ZNF516, ZNF804A
FIB	60	AC008440.2, AC112217.2, **ADAM33,** ADH1B, AIM1, AK5, APCDD1, APOD, AQP9, BMPER, C9orf21, CCBE1, CCDC102B, CCRL1, CDON, CH25H, CLIC2, COLEC12, CYP7B1, DKK2, DNM1, GLDN, GLI3, GRPR, GTSF1, IGSF10, KIAA0802, **KIT,** LRRC15, MASP1, MBNL3, **MMP27,** OMD, OR1H1P, OR1Q1, OSR2, PAX3, PDGFRL, PHACTR3, PLEKHG1, PLXNC1, PRLR, RCAN2, ROBO2, RP11-392O17.1, RXFP1, SECTM1, SEMA3D, SIPA1L2, SLC9A9, STK32B, SVIL, TDO2, TFAP2C, TGFBR3, THBS4, THRB, **TWIST1, TWIST2**

Gene symbols in bold correspond to relevant genes that are commented in the discussion

Correspondence or overlapping between DE gene sets obtained from the two approaches presented (i.e. the meta-analysis set derived from public data series and our *de-novo* set), reaches a percentage of 47% for up-regulated genes in BM-MSC against HSPC.

### Cytokine-receptor mapping based on expression patterns

Tissue-repair processes mediated by MSCs has been proven in many cases as an effect of secreted growth factors, cytokines, and other signalling molecules [14]. Cytokines have the capacity to trigger the signalling cascades from cell-to-cell within a tissue. The data presented in this work allow us to study the expression patterns of cytokines along populations of tissue-MSCs.

**Fig. 5:**
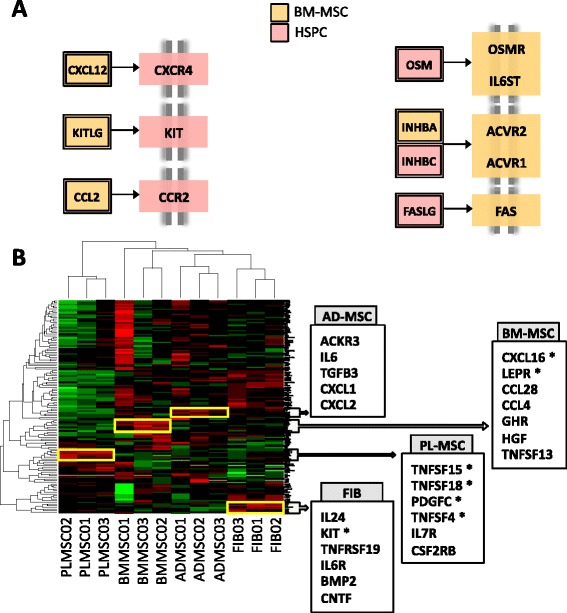
Cytokine expression patterns of MSCs. **a** Cytokine interactions between BM-MSCs and HSPCs. Differentially expressed cytokines in BM-MSCs (*yellow*) and HPCs (*red*) were coloured over the interaction map, highlighting the potential interactions established between the two cell types in the bone marrow. A complete mapping over the cytokines KEGG pathway can be seen on Additional file 8. **b** Heatmap clustering of microarrays samples based on the cytokinome intensity patterns. A gene cluster has been selected per stromal cell class. Stars denote double positive cytokines also found in the intersection of the corresponding differential expression contrasts

Within the bone marrow niche, cytokines forge the interactions within the two major inhabiting stem cell populations: hematopoietic and stromal. Using our meta-analysis data frame, we have looked for cytokines among the genes up-regulated in BM-MSCs and in HSPCs, and mapped them over the cytokine to cytokine-receptor interactions (defined by KEGG). In Fig. 5a, several mesenchymal-to-hematopoietic crossed interactions from the KEGG pathway have been represented (whole mapping is included in Additional file 8).

The search across the meta-analysis dataset yielded 28 cytokine genes among 2,124 BM-MSC up-regulated genes (hypergeometric enrichment *p*-value = 0.0099). Within the same dataset, 52 cytokines were found among 2,624 HSPC up-regulated genes (hypergeometric enrichment *p*-value = 4.56 × 10e-09). Interestingly, HSPCs showed expression of a greater bunch of cytokines than MSCs, which were named cytokine ‘drugstores’ [15]. Previously described critical interaction pairs, like CXCL12–CXCR4 or KITLG–KIT, were present in our interaction map. From the set of 28 BM-MSC cytokines found in the meta-analysis, 11 appeared also differentially expressed in the comparison of BM-MSCs versus HSPCs in our *de-novo* study. These cytokines are: ACVR1, BMPR1A, BMPR2, CCL2, IL13RA1, OSMR, PDGFRB, TGFBR2, TNFRSF11B, TNFRSF12A and VEGFC.

Going further, we wanted to identify the potentially tissue-specific cytokines of each stromal cell subtype. We applied a hierarchical semi-supervised clustering analysis over the cytokine panel using the expression profiles of the 12 stromal samples from the *de-novo* dataset. Figure 5b accounts for the 50% most variable cytokines across all sample classes. Clusters showing predominant expression in just one class of stromal cells, according to their tissue of origin, have been highlighted. For the bone marrow, cytokines CXCL16 and LEPR lead the profile of a 7-gene cluster. Associated to the placental tissue, the cytokine pattern highlights TNFSF4, 15 and 18, and PDGFC. Finally, KIT and BMP2 genes resulted over-expressed factors in the skin-derived fibroblasts.

### Mesenchymal lineage expression core signature

**Fig. 6:**
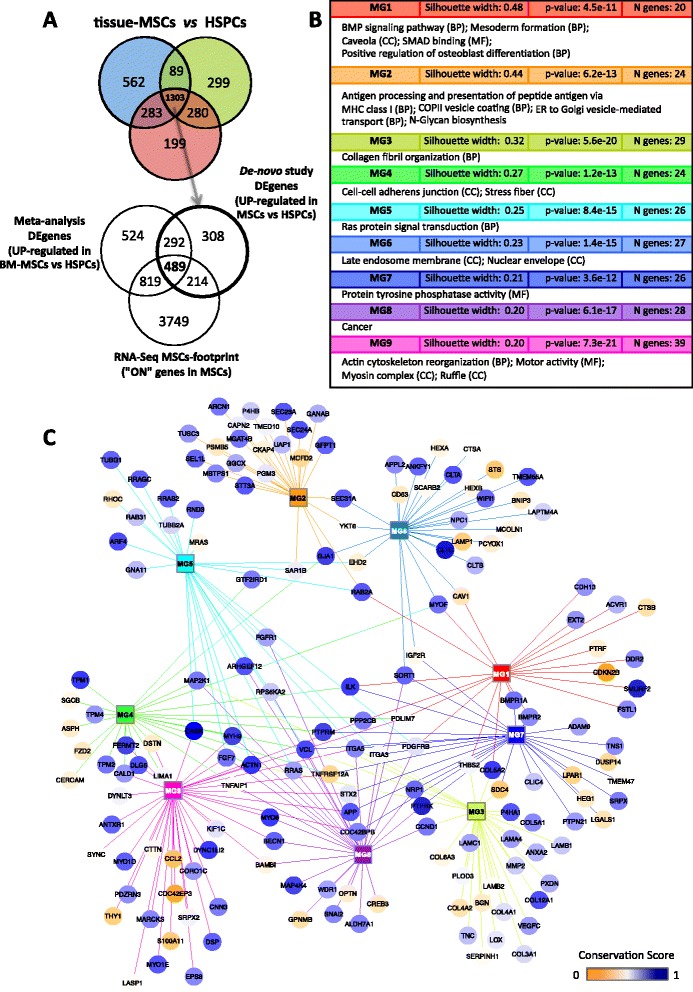
Mesenchymal lineage signature. **a** Venn Diagrams showing the cross-flow of differentially expressed genes from the three approaches presented that contrast MSCs to HSPCs. Colour code for tissue-MSCs: BM (*red*), PL (*blue*), AD (*green*). **b** Summary table of enrichment in biological terms (MG = Metagroup): Metagroup refers to a functional cluster that gathers functionally associated genes from the mesenchymal lineage signature. The same MG colour code is applied in the network. The value *Silhouette width* measures the closeness between biological terms within each metagroup. The higher the *Silhouette width,* the more closely related are the metagroup terms. *P*-values give the enrichment significance to each metagroup. **c** Functional network. Ball-nodes represent genes. Links represent functional association between genes based on shared biological annotations. Squared nodes represent the metagroups of genes sharing functional annotations. Conservation information per gene (based on exonic sequences) has been implemented under a *yellow-blue* scale, from less to highly conserved scores, respectively

The hematopoietic cellular element (HSPCs), present in both transcriptomic datasets studied, is the common non-mesenchymal element of the cellular populations analysed. We have used this out-of-lineage population as contrast group to generate a mesenchymal lineage expression signature. Genes found up-regulated in each comparison of the tissue-MSCs (AD, BM and PL) against the HSPC set were matched together (see Venn diagram in Fig. 6a). Common genes up-regulated in all MSCs versus HSPCs were selected, obtaining 1,303 shared by the three tissue-MSCs included whithin the *in-house* dataset. From this signature, 489 genes overlapped with the BM-MSC up-regulated genes from the meta-analysis and with the previously characterised MSC gene footprint defined by our group using RNA-Seq [16]. Therefore, we can consider this set of 489 genes as the most conservative core of differentially expressed genes in MSCs, i.e. the genes that compose a well defined “mesenchymal lineage expression signature” (the complete list is included in Additional file 9).

As significant elements that support the value of the signature, we can highlight some of the mesenchymal specific genes included in it: stromal standard biomarkers THY1 (CD90) and NT5E (CD73), transcription factor SNAI2 (that is an epithelial-mesenchymal transition regulator), several collagen molecules involved in the stroma (COL3A1, COL4A1, COL4A2, COL5A1, COL5A2, COL6A3, COL12A1), and the BMP receptors (BMPR1A, BMPR2) involved in the differentiation and specification of mesenchymal precursor cells. Moreover, together with SNAI2, this MSC lineage signature includes 12 other transcription factor genes: ARID5B, CREB3, EPAS1, FHL2, GTF2E2, GTF2IRD1, ID3, LMO7, TAF13, TEAD3, TULP3, and ZNF532.

Biological processes prompted by these 489 genes were unravelled applying functional enrichment analysis tools. The *GeneTerm-Linker* functional analysis tool provided a non-redundant clustering of biological terms to which the signature genes were annotated. The table in Fig. 6b shows a summary of the results (the complete table is found in Additional file 10). Nine clusters of enriched biological terms and functions (called metagroups and labelled as *MG*) were found. Significantly, functions related to *BMP signalling, osteoblast differentiation, mesoderm formation*, *collagen fibril organization and extracellular matrix structure*, appeared among the biological enriched annotations.

To validate the generated MSCs signature, we performed another principal component analysis over our *de-novo* produced dataset of 15 microarrays (9 tissue-MSCs, 3 HSPCs and 3 Fibroblasts) using only the 489 genes included in the mesenchymal lineage expression core signature (results presented in Additional file 11). The outcome of this PCA shows a cumulative variance explained by the genes in the first 3 components of: PC1 78.79%, PC2 91.76%, PC3 94.20% (using the samples as variables and represented as a *Biplot* in Additional file 11 A); and a cumulative variance explained by the samples in the first 3 components of: PC1 84.04%, PC2 88.51%, PC3 91.91% (using the genes as variables and represented as a 3D plot in Additional file 11 B). These two figures show that the separation between hematopoietic and mesenchymal lineages is more strongly marked compared to the previous PCA done with the whole transcriptomic signal using the 264 meta-analysis microarray dataset (Fig. 2b and c). The plots also show that the PCA based on the 489 genes of the MSCs signature provides much better separation between the cell subtypes than the PCA based on the whole gene expression signal of the 15 exon microarray samples of this study (Fig. 3a and b). Clusters are better defined now, and PC3 allows an evident separation of the stromal cell samples, including a 91.9% explained cumulative variance. Moreover, the fibroblast samples come apart in a cluster separated from the tissue-MSC samples. This is an interesting new perspective, considering that the analyses previously performed with the whole transcriptome in different sample sets were not able to provide such clear segregation.

### MSCs functional gene network

We built a functional network founded on the metagroups recruited from the enrichment analysis (Fig. 6b, c) for better analysis and visualisation of the meaningful associations between the 489 MSC genes. Genes are linked to the metagroups in which they are annotated; in other words, the graph displays clusters of genes sharing similar biological annotations and functional roles [17]. Genes (circular nodes) linked by edges to metagroups (squared nodes) reflect the membership to a specific functional group and show the sharing of common biological roles with other genes. In this bipartite network, the metagroups act as hubs denoting integrative roles within the mesenchymal core signature. Some metagroups can share many genes with others, but other metagroups are more exclusive. In this way, a general overview of this functional gene network (Fig. 6c) indicates that the first metagroups (MG1, MG2 and MG3, with higher silhouette width > 0.30), include more specific mesenchymal-stromal functions (e.g. *mesoderm formation*, *collagen organization, cell-to-cell adhesion*), and appear more detached to the rest of functional metagroups. By contrast, MG7 and MG8 (showing silhouette width < 0.25) are more promiscuous, with greater overlap and many edges cross-linking multiple genes. The functional annotations for these metagroups are more diffuse or general (e.g. *cancer*, *protein tyrosine phosphatase activity*).

Regarding the genes, some are included in many functional metagroups, as ITGA5 (integrin alpha-5, CD49e fibronectin receptor), revealing a more central role. In fact, this gene is connecting five metagroups in the network: MG3, 5, 7, 8 and 9. CD49e fibronectin receptor is known as essential for the cell attachment to the extracellular matrix (ECM); but, intracellularly, it is involved in the formation of adhesion complexes with cytoskeleton proteins and in the activation of kinases that regulate signaling of growth, division, survival, differentiation, migration or apoptosis. The multiple regulatory functions that this protein integrates endorsed the network position that it presents. Likewise, another gene central element is the mitogen-activated protein kinase kinase (MAP2K1), which is found as acting as connection point for several metagroups: MG3, 4, 5 and 8. This enzyme lies upstream of the MAP kinases pathway and is essential for signal transduction. Other genes connecting more than two metagroups in the network are: ACTN1, GJA1, ILK, MYOF, PDGFRB, PDLIM7, RAB2A, SORT1, VCL.

To add another layer of information, we also investigated whether genes connecting separate functions within this mesenchymal lineage network (i.e. genes that are more central in the network) might present higher or lower evolutionary conservation. To achieve this, we incorporated sequence conservation information, based on the exonic coding regions, to each gene of the functional network. Additional file 12 contains the calculated conservation scores of the complete list of genes. In the network, the conservation is displayed as the node colour, scaled from orange to blue, from least to most conserved genes, respectively. As a whole, we observed that the less conserved genes (i.e. genes more recent in evolution) belong only to one functional metagroup (e.g. CDC42EP3, CDKN2B, SDC4, STS), but the most conserved are usually connected to several functional metagroups (e.g. CASK, ILK, GJA1). This is a trend consistent with the idea that conserved genes may be more multi-functional, since they are more ancient and can acquire new roles along evolution. For example, we identified CASK as a highly conserved gene that links MG5 and MG9. This gene encodes a membrane-associated calcium/calmodulin-dependent guanylate kinase (a serine kinase). The known functions of CASK indicate that it is a scaffold transduction protein located at synapses in the brain that contributes to neural development, maintains the morphology of neuron dendritic spines, and regulates cellular interactions at the presynaptic site. It is clear that this protein detected in MSCs will have other functions in the stromal niche, showing that it is a multi-functional gene. Among the less conserved genes we found, for example, CDKN2B, CDC42EP3, CCL2, LAMP1, STS and THY1 (CD90). The presence of the MSCs marker CD90 in this list might indicate that more recently evolved genes may also be more specific within the mesenchymal signature.

### MSCs gene coexpression network

The network presented in Fig. 6c corresponds to a functional gene network derived from the enrichment analysis perforrmed with the 489 gene signature obtained for the MSCs. To complement this network analysis, we also built a coexpression network derived from the transcriptomic data, i.e. from the expression values of the 489 genes along the exon arrays in 15 samples of the different cell types studied (that is, an experimentally-driven network). We calculated the coexpression using the *Pearson* correlation, and we applied a stringent cutoff to select the pairs with correlation coefficient *r* ≥ 0.95 to identify the most correlated gene pairs. We provide an Additional file 13 presenting this network. In this analysis, we highlight the genes that correspond to known CD markers (16 CDs) and to transcription factors (11 TFs) to better illustrate possible interesting links. Some genes like the receptors FGFR1 and PDGFRB are highly connected in both networks (Fig. 6c and Additional file 13), indicating that they are relevant to the nature of the MSCs. To allow a more detailed exploration of the coexpression network built, we provide a Cytoscape file that includes all the interactions derived from the correlations and it incorporates 447 genes and 9969 interactions (Additional file 14).

### Functional analysis of the genes expressed in specific subtypes of MSCs

Figure 6a presents the genes detected as commonly overexpressed (up-regulated) in the contrast of all the MSCs versus the HSPCs, which provided an intersection set of 1303 genes. As a contrast to this common set, we also investigated the genes and gene functions that were specific to each subtype of MSC. To do this, we selected the genes found overexpressed in each tissue-MSC when compared with the other stromal cell types. These comparisons provided: 281 genes for AD-MSCs, 421 genes for BM-MSCs and 531 genes for PL-MSCs; and correspod to the gene lists derived from the union of the data represented in the *Venn* diagrams in Fig. 4f. These three gene lists were submitted to a functional enrichment analysis to identify their biological meaning using DAVID functional enrichment tool (Methods). A first view of these results (included in Additional file 15), shows that these lists include genes that correspond to a set of common biological terms: *signal peptide*, *extracellular matrix*, *glycoprotein* and *secreted protein*; that correspond to functions that are enriched in all the tissue-MSCs but not in fibroblasts, indicating that these cells have a tendency to produce much signaling and secreted proteins, often present in the extracellular matrix. Looking for more specific functional profiles, we observed that AD-MSCs and BM-MSCs showed a strong enrichment for terms like *skeletal system development* or *embryonic skeletal system development*, that indicate a closer association with bone differentiation and skeleton maintenance. We also observed that homeobox genes (HOXA, HOXB, HOXC, SIX1, SIX2) were highly present within these functional annotations, together with other genes like: ACVR2A, ALPL, GAS1, MGP, TGFB2, TGFB3, TGFBR2. Several of these genes are related to calcium homeostasis and bone morphogenesis or to growth and differentiation in specific cellular contexts; highlighting the functional similarity of the MSCs coming from bone marrow or adipose tissue.

### Overlapping with other reported MSCs gene signatures

Once we analysed human MSC expression profiles and outlined several stromal cell-, tissue- or lineage-associated gene signatures, we searched public databases and publications to find which genes were previously reported using similar approaches. In Tables 4 and 5, several human MSC transcriptomic studies have been summarised, and the reported MSC gene signatures ordered and compared to the outcomes of our study. The overlapping proportions with different datasets are variable, but the similarity is rather significant, being aware of the technological chages along time, the differences in expression platforms as well as the use of different data handling protocols. In this way, we found several genes included in our MSCs lineage signature that repeatedly appeared in gene lists produced in similar published works. Table 4 presents a featured overview of the overlap of the MSCs lineage signature proposed with other studies (highlighting in bold in the last column the genes that appear in more than one list). Some of the genes in these genesets are already known as phenotypical hallmarks of the mesenchymal identity, like the biomarker CD73 (NT5E). Others are known actors in epithelial to mesenchymal transitions: IGFBP3, DDR2, LOX, LOXL2 [18]. Another gene present in several of the published lists derived from MSC studies is SMURF2, an E3 ubiquitin ligase that seems to cooperate with SMAD7 in preventing myofibroblast differentiation via TGFbeta receptor destabilisation [19]. RHOC is another interesting gene product that encodes a member of the RHO family of small oncogenic GTPases, and promotes reorganisation of the actin cytoskeleton, thus regulating cell shape, attachment and motility. Finally, PLOD2 is an enzyme that catalyses the lysyl-hydroxylation in collagen peptides, critical for the stability of inter-collagen crosslinks. All these biological functions are deeply related to the stromal cells niche supporting the value of the MSCs lineage signature found.

**Table 4:** Review of gene signatures previously published for MSCs and comparison with the proposed MSC lineage signature of 489 genes

Reference	Organism	Cells types in contrast	DE features	Statistical significance threshold	Observations	Overlap of the proposed MSCs lineage signature with other studies
Tsai et al. 2007	*Human*	Four MSC types (BM, AF, AM and CB) vs 6 whole sampled tissues (brain, heart, lung, liver, kidney, and muscle)	47 genes commonly UP in MSCs, forming a core signature	>4-fold change (*p* < 0.000005)	Human U133A GeneChip (Affymetrix) 22,000 probe sets that span 14,500 genes	21 genes in the MSC lineage signature: **ACTN1**, **ADAM9**, **ANXA5**, **CALU**, **CAV1**, **CTGF**, **KDELR3**, **MCFD2**, **NNMT**, NT5E (CD73), **LOX**, **LOXL2**, PHLDA1, **PLOD2**, **S100A11**, **SERPINE1**, **SMURF2**, **TAGLN**, TIMP1, **TPM4**, UAP1
Kubo et al. 2009	*Human*	BM-MSC vs differentiated cells (FIB included)	148 genes UP in BM-MSC	>2-fold change	Affymetrix Human Genome U133 plus 2.0. 54,000 probe sets / 38,500 genes. Differentiated cells include FIB, osteoblasts, adipocytes and chondrocytes.	14 genes in the MSC lineage signature: **ADAMTS5**, **CCND1**, FHL2, GDF15, **IGFBP3**, **LOX**, **LOXL2**, MAP4K4, **MCFD2**, SH3RF1, SLC16A4, **SMURF2**, UGCG, VEGFC
Jääger et al. 2012 [41]	*Human*	AD-MSC or FIB vs their lineage derived cells	211 genes UP versus cells differentiate to 3 lineages	ANOVA (1% FDR)	AD-MSC and FIB were in vitro differentiated towards osteogenic, chondrogenic and adipogenic lineages.	16 genes in the MSC lineage signature: CRISPLD2, HTRA1, IGFBP5, LAMB1, MARCKS, **NNMT**, PSAT1, REXO2, RPS27L, **S100A11**, SARS, **SERPINF1**, TMEM165, TMEM47, VAMP3, WARS
Jääger et al. 2012 [41]	*Human*	AD-MSC or FIB vs their lineage derived cells	333 genes DOWN versus cells differentiate to 3 lineages	ANOVA (1% FDR)	AD-MSC and FIB were in vitro differentiated towards osteogenic, chondrogenic and adipogenic lineages.	38 genes in the MSC lineage signature: **ACTN1**, **ADAM9**, **ANXA5**, ARF4, C1R, C1S, **CALU**, **CAV1**, **CCND1**, CD63, CKAP4, CLIC4, CORO1C, CTGF, CYB5A, DDAH1, **DDR2**, DSP, ELL2, FRMD6, FTL, HEG1, HEXA, HNMT, IER3IP1, ISLR, **KDELR3**, LAMA4, LAMC1, LASP1, LMO7, MYH9, NRP1, NUPR1, PTRF, **SERPINE1**, SRPX, TUBB6
Jääger et al. 2012 [41]	*Human*	AD-MSC or FIB vs their lineage derived cells	genes UP or DOWN in distinct contrasts versus cells differentiate to 3 lineages	ANOVA (1% FDR)	AD-MSC and FIB were in vitro differentiated towards osteogenic, chondrogenic and adipogenic lineages.	10 genes in the MSC lineage signature: **IGFBP3**, MT1E, MYOF, NAV1, NTM, PDLIM7, PTPN21, **RHOC**, SH2D4A, **TPM1**
Pedemonte et al. 2007 [30]	*Mouse*	BM-MSCs vs any given tissue or cell type of the 12 included (brain, heart, skeletal muscle, liver, kidney, lung, dendritic cells, ESCs, MEFs, NSCs, HSCs, T-cells)	403 genes (translated into 249 human orthologs)	F-test *p* < 0.0001	Affymetrix Mouse Genome 430 2.0 arrays, covering 39,000 transcripts. 7 BM-MSC samples against 486 publicly available samples from different origins.	54 orthologs genes in the MSC lineage signature: **ADAMTS5**, ANTXR1, ANXA2, ASNS, BGN, C6orf89, CALD1, CDC42EP3, CNN3, COL3A1, **COL4A1**, **COL5A1**, COL5A2, COL6A3, CSRP1, DAP, DBN1, **DDR2**, DKK3, DPYSL3, ERRFI1, FGF7, FSTL1, FZD2, GJA1, GPR124, HTRA1, **KDELR3**, LGALS1, **LOX**, MMP2, MRC2, NID2, **NNMT**, NUPR1, OSMR, PDGFRB, PHLDA3, **PLOD2**, PLS3, POSTN, PSAT1, PYCR1, RCN1, RHOC, RNASE4, TNFRSF12A, TPBG, **TPM4**, TSPAN6, **SERPINF1**, **SERPINH1**, SNAI2, SPARC

Genes appearing in more than one published signature are highlighted in bold

**Table 5:** Comparison of fibroblastic signatures previously published against our differentially expressed genes in equivalent contrasts

Reference	Organism	Cells types in contrast	DE features	Statistical significance threshold	Observations	Overlap of genes detected in FIB versus MSCs signatures of different tissue origen studied in this work
Wagner et al. 2005 [7]	*Human*	HS68-FIB against all tissue MSCs (BM-MSC-M1; BM-MSC-M2; AD-MSC-M1; CB-MSC-M3)	30 genes UP in FIB (based on 206 ESTs)	> 4-fold change	Human Transcriptome Microarray representing 51,144 different cDNA clones of the UniGene set RZPD3 (two colour arrays). M1, M2 & M3 refer to different culture conditions.	9 genes UP in FIB vs MSCs: APCDD1, CCRL1, **KIT**, **MMP1**, MMP3, MOXD1, PSG3, PSG9, TBX5,
Jääger et al. 2012 [41]	*Human*	AD-MSC vs FIB	119 genes UP in FIB	ANOVA (5% FDR)	Multiplex mRNA-sequencing	14 genes UP in FIB vs MSCs: ANPEP, CDC25B, CLDN11, CTSK, CXCL12, DNM1, IGFBP3, **MMP3**, PBX3, S100A4, SLIT2, STEAP1, TRAF3IP2, VIT
Wagner et al. 2005 [7]	*Human*	All tissue MSCs (BM-MSC-M1; BM-MSC-M2; AD-MSC-M1; Cord Blood MSC-M3) vs HS68-FIB	25 genes UP in MSCs (based on 47 ESTs)	> 2-fold change	Human Transcriptome Microarray representing 51,144 different cDNA clones of the UniGene set RZPD3 (two colour arrays). M1, M2 & M3 refer to different culture conditions.	4 genes DOWN in FIB vs MSCs: GPC4, **HOXA5**, **PLOD2**, TM4SF1
Jääger et al. 2012 [41]	*Human*	AD-MSC vs FIB	59 genes UP in AD-MSC	ANOVA (5% FDR)	Multiplex mRNA-sequencing	13 genes DOWN in FIB vs MSCs: BGN, CDH2, GGT5, ID3, **COL4A1**, **COL5A1**, COL11A1, KRT18, **LOXL2**, NDFIP2, TAGLN, TNS1, **TPM1**

Genes appearing in more than one published signature are highlighted in bold

As a contrast inside the stromal lineage, we also found genes that appear to be specifically associated to MSCs but not expressed in FIBs (Table 5). These genes show interesting roles, for example: the family of HOX regulatory factors (HOXA5) involved cell differentiation, cell adhesion/migration and cellular development; or several collagen molecules highly expressed in MSCs (COL4A1, COL5A1). These contrasts also reveal specific up-regulated genes just in FIBs, like KIT, and genes of the MMP family (MMP1 and MMP3) that were also found in the set of 60 genes that we assigned to be fibroblast-specific (see Table 3 and Fig. 4f).

## Discussion

### Mesenchymal stromal/stem cells phenotyping

The term “mesenchymal stromal/stem cell” summarises a complex cellular entity that can be isolated from diverse tissues in the human body, sharing similar morphology, growth conditions, cytometric patterns and in vitro differentiation capacity. Despite these parameters that have been agreed and standardised for MSCs [20], a detailed biomolecular profiling of this cell type is lacking; and it is common to find fluctuations in the identification of these cells and in the reports of gene or protein markers assigned to them. In fact, many research articles about their specific phenotypic markers have been published (as summarised in [21]). Multiple efforts have been undertaken to discover biomarkers that allow homogeneous isolation of these cells. Despite these efforts, the field is still open to deeper molecular characterisation. In this work, we have conducted a broad genomic-based approach that provides a data-driven characterisation of the genes activated in the MSC phenotype, reinforcing some already proposed knowledge and providing new insights inaccessible to previous procedures (Tables 4 and 5).

### Previous studies on primary stromal cells and the bone marrow niche

Several relevant publications [22–25] have contributed to the characterisation of the cellular and molecular components of the bone marrow niches (i.e. endosteal osteoblasts, perivascular stromal cells, endothelial cells, Cxcl12 abundant reticular cells, Lepr^+^ stromal cells, Nestin^+^ mesenchymal progenitors) where the HSCs reside, proliferate, mobilise or differentiate. The main consideration we need to be aware of is the fact that all this information comes from experiments in vivo in “mice”. Therefore, the advances of these reports cannot be directly extrapolated to the human bone marrow microenvironment. However, these studies are of broad interest to contextualise previously described interactions from mice into the human hematopoietic niche. We discuss these and other related studies that provide information about the transcriptomic profiling of MSCs.

The highly expressed chemokine CXCL12 is a potent attractant and retainer of both HSCs and MSCs, critical for maintaining hematopoietic stem and progenitor cells (HSPCs) in a quiescent state. CXCL12 abundant reticular cells (named “CAR cells”) were first shown to guide a depletion of HSCs when the Cxcl12 gene was knocked-down, as well as severely impair the adipogenic and osteogenic capacities of these stromal cells [26]. Moreover, deletion of CAR cells reduces the number of B-lymphoid progenitors [27]. This HSC niche-related cytokine was shown as expressed in CD146^+^ perivascular mesenchymal cells in humans [11], with capacity to generate osteoblastic cells and promote HSC maintenance.

Niche cells for HSC maintenance are marked by Nestin (Nes), an intermediate filament protein found in self-renewing mesenchymal stem cells (MSCs) [22]. Nes^+^ cells express very high levels of Cxcl12. Futhermore, PDGFRα^+^/Sca1^+^ (PαS) cells have been suggested to comprise bone marrow MSCs [28]. In fact, the frequency of CFU-F, a hallmark of MSCs, in Prx1^+^ PαS cells is much greater than that reported for Nes^+^ cells, suggesting that Prx1^+^ cells are indeed mesenchymal progenitors [24].

Apart from highly expressing CAR cells and MSCs, other stromal cells committed to osteogenic lineage or endothelial cells also express Cxcl12, though in lower levels. Several studies have evaluated the functional impact of Cxcl12 removal in different stromal populations of the niche [23, 24, 29], thus defining distinct specialised niches for HSC maintenance, HSC retention, and the generation of certain lymphoid progenitors. Deletion of Cxcl12^+^ in osteoprogenitors (*Sp7* or *Osterix* positive cells) causes a significant reduction of T- and B-cell production, and fewer early lymphoid progenitors. Leptin receptor (Lepr) is present in perivascular sinusoidal stromal cells that express high levels of stem cell factor (SCF), which was previously reported as essential for HSC maintenance [23]. Lepr is also involved in a novel hematopoietic pathway that is required for normal lymphopoiesis. Another role has been suggested for Cxcl12, derived from Lepr-marked cells, that was related to the retention of HSCs rather than to their maintenance. This role comes from the observation that reduction of Cxcl12 expression within the sinusoidal stromal compartment does not alter HSC or progenitor numbers, but induces the mobilisation of HSCs and progenitors to the spleen and peripheral blood.

Through analysis of the data generated by our work that showed overexpressed genes in different human tissue-MSCs compared to HSPCs, we can derive several interesting observations related to the markers identified in mice studies: (i) we show the presence of CXCL12, LEPR, KITLG in the specific expression of BM-MSCs; (ii) CXCL12, but not LEPR, was also overexpressed in AD-MSCs; (iii) PL-MSCs did not show overexpression of CXCL12 or LEPR; (iv) PDGFA, PDGFC, PDGFRB and PDGFRA are generally expressed in MSCs from all the tissues studied; (v) RNA-Seq data showed a strikingly higher expression level of the Nestin gene (NES) in human PL-MSCs compared to BM-MSCs [16].

### Consistent finding of CDs to mark the MSCs

The study of MSCs in primary cultures implies the growth in heterogeneous populations with unknown proportions of differently committed cells. Thus, expressed genes may strongly vary from culture to culture, setting the reason why signature or biomarker characterisation is sometimes irreproducible. In other words, it produces an increased rate of false positives in differential expression and a reduced true positive rate. The present work addressed these problems applying several strategies to solve them: (i) integration of multiple datasets including a large number of samples of our interest collected from different studies; (ii) construction of an adequate cell biology framework with a well-distilled and contrast-minded sample cell types selection; (iii) application of robust re-sampling techniques to find stable and reproducible signals. In this way, DE genes yielded by the multiple analyses produced steady and reliable expression signatures. The genes selected may not be the most strongly expressed for a sample category in a single test. Instead, they presented a rather constant differential expression pattern along the samples, significantly surpassing the iteraction tests. Thus, despite possible variability in the samples, genes presenting subtle changes stable along samples, were preferably captured. Proof of the methodology success is given by the retrieval of official MSC CD marker genes (i.e. CD73, CD90, CD105) in the BM-MSC signature (Table 1).

CD146 (melanoma cell adhesion molecule MCAM, a determinant of hematopoietic perivascular niches) stands steadily over-expressed in BM-MSC cultured populations when they are repeatedly compared to hematopoietic progenitors and dermal fibroblasts. Analogous expression profiles had been observed by other molecular techniques and independent signatures [9]. Covas et al. [9] reported a close transcriptomic relationship between CD146^+^ perivascular cells from BM-MSCs; also segregating them from fibroblasts, which did not expressed it. Moreover, it has been observed that therapeutically applied BM-MSCs preserve a population of CD146^+^ perivascular cells. Another 7 CDs out of the 28 listed in the BM-MSC signature have been used as molecular markers for MSC and other stem cells (CD13, CD49e, CD58, CD73, CD90, CD105 and CD140b) (updated from Calloni et al.) [21]. Considering the correlation with previous knowledge, novel interest may be focused on the other highly confident CDs presented in this work. These molecules may compose the distinctive body of CDs available for BM-MSCs (Table 1).

### The fibroblast in the stromal cell context

Pedemonte et al. published an extensive transcriptomic study using mouse MSCs and related cell types [30]. A collection of 486 microarrays from many tissues and cell types allowed the allocation of the mesenchymal lineage in a comprehensive panel of lineages. Interestingly, the closest cell type that clustered with BM-MSCs was the fibroblastic type (specifically MEFs, the murine embrionary fibroblasts). A transcriptomic-based study was also reported by Chang et al., that provided an unsupervised comparative analysis and clustering of human fibroblastic populations obtained from different sites of the human body [8].

The stromal cells studied here presented clear differences between them, attributed mainly to their tissue-linked origins. Differences observed between the fibroblasts and the MSCs categories are not large, although the fibroblasts are considered the stromal type with the highest expected degree of differentiation. This means that the MSC populations that we are investigating, in terms of their trancriptome, are a much closer cell type to the fibroblast than initially expected. Our flow cytometry data also supported the similar expression of CDs between MSCs and fibroblasts. This similarity has been previously reported [31, 32] and Haniffa et al. even proposed that fibroblast and MSCs are functionally equivalent [32]. Controversial results about the differentiation potential of fibroblasts toward adipoblasts, chondroblasts or osteoblasts, keep the fibroblast cell under questioning [33–35]. The work from Alt et al. [35] encountered human skin fibroblast cultures capable of differentiating and forming CFUs in vitro. As the separation between MSCs and fibroblasts seems small, we found more fruitful the contrasts of MCSs isolated from different tissue origins against HSPCs to extract a mesenchymal multilineage defining gene catalogue.

### Mesenchymal lineage signature

We collected the gene sets found to be up-regulated in MSCs relative to hematopoietic stem cells from all of the performed contrasts: (i) the three contrasts from the tissue-specific MSCs versus HSPCs (presented in Fig. 6a), and (ii) the 150 re-sampled contrasts obtained through the meta-analysis approach of BM-MSCs versus HSPCs (Additional file 6, contrast 1). These gene sets were analysed, to find the overlapping, and also compared with the genes found “ON” in the MSCs footprint (obtained with RNA-Seq) [16]. Following this integrative approach, we were able to delimit an expression core of 489 common genes that support and maintain the multipotency of the mesenchymal lineage. The functional enrichment of this MSC gene signature indicates a strong osteogenic association of the genes, that has been postulated as the most frequently chosen path of the cellular commitment programs for MSCs. Moreover, it has been observed that the osteogenic potential capacity is lost the last when a progressive in vitro model of multipotency is established [34, 36].

Charbord and colaborators [25] defined a large gene signature (including 481 mRNAs) whose expression is associated to the support of the hematopoietic niche. This signature inluded most of the aforementioned genes related to the bone marrow niche. We compared the Charbord signature with the up-regulated genes detected in each of our tissue-MSCs (derived from the comparisons versus HSPCs). The number of genes matching each overexpressed gene-list was: 121 genes in AD-MSCs, 125 in BM-MSCs, and 119 in PL-MSCs. This result indicates that the BM-MSCs are the closest to the described hematopoietic niche, and are closely followed by AD-MSCs and PL-MSCs. However, a recent publication by Reinisch and colaborators [37] stated that only BM-MSCs, and not other tissue-MSCs, were capable of developing an endochondral ossification of human cells in a mice model that was preceded by the formation of a functional hematopoietic niche. Our data may be compatible with this notion if we consider that just 4–6 genes can make a significant difference, although proper experimental testing with these genes will be the only way to prove the specificity of the BM-MSCs versus other subtypes.

### Some genes derived from the network reconstruction

Aided by the graphical view of a network, genes that behave like hub connectors of functions could be uncovered, opening the door to new potential regulators of the mesenchymal system. For example, SORT1 gene (sortilin 1) occupies a hub position between three functional metagroups that connect vesicle trafficking, tyrosine phosphorylation signalling and molecular processes usually altered in cancer (Fig. 6c). The protein encoded works as a sorting receptor in the Golgi apparatus required for protein transport to the lysosomes. Interestingly, it has been shown that SORT1 promotes mineralisation of the extracellular matrix during osteogenic differentiation by scavenging the extracellular lipoprotein lipase produced by adipocytes [38]. This gene might be an actor of the fine equilibrium between differentiation paths.

### Transcription factors

Transcription factors (TFs) are the principal regulators of fate decision. In the MSC lineage signature we found 13 TFs: ARID5B, CREB3, EPAS1, FHL2, GTF2E2, GTF2IRD1, ID3, LMO7, SNAI2, TAF13, TEAD3, TULP3, ZNF532 (Tables 4 and 5). Two of these TFs have enrichment in transcription factor binding sites (TFBS) in the promoters of a significant number of targeted genes within the 489 MSC signature: CREB3 (301 targets) and TEAD3 (210 targets). TF SNAI2 has been largely documented as a central booster of mesenchymal phenotype, as occurs along EMT processes (epithelium-mesenchymal transitions) [39]. SNAI2 transcriptionally represses expression of E-cadherin, but may act as an activator depending on the biological context. Recently, it has been intricately related to osteoblast maturation through interaction over RUNX2 and CXCL12 promoters [40]. Another nine factors are accompanying SNAI2 into this signature, including ID3 (inhibitor of DNA binding). ID3 is a helix-loop-helix (HLH) protein that can form heterodimers with other HLH proteins, thus preventing them to bind their target DNA regulatory regions. It is known that ID3 inhibits the skeletal muscle and cardiac myocyte differentiation by seclusion of E2A-complexes from E-box enhancer of muscle creatine kinase. In our data, ID3, together with PAWR (or PAR4), was robustly overexpressed in BM-MSCs relative to fibroblasts. PAWR is a pro-apoptotic WT1-interacting protein that functions as a transcriptional repressor. PAWR induces apoptosis by activation of the FAS pathway, and co-parallelly by inhibition of NFKB in certain cancer cells, specifically prostate cancer. Intriguingly, SNAI2, ID3 and PAWR have been generally discovered by their function as transcriptional repressors.

### Tissue-specific MSC genes

Differences between stromal populations derived from various tissues are becoming more evident, and represent a source of heterogeneity within the mesenchymal phenotype. All stromal cells are essentially able to differentiate into the three mesenchymal fates in vitro, although they do not follow the same molecular paths. The cells seem to keep expression “memory” of source-specific genes that travel along during the differentiation process [41]. Recent transcriptomic studies developed in murine endothelial cells from a plethora of tissues have also manifested the tissue-specificity of their molecular signatures, heterogeneity that is explained by their function in maintenance and regeneration of the different microenvironments [42].

Using our *in-house* dataset to contrast tissue populations against each other, we were able to describe a repertoire of tissue-specific genes from four tissue stromal populations: bone marrow, placental and adipose tissue MSCs also in addition to dermal fibroblasts. Curiously, AD-MSC showed the lower numbers of differentially expressed genes in the comparisons with the other MSCs, showing the smallest number of exclusively adipose tissue genes when compared to the rest of the stromal cells (Venn Diagrams in Fig. 4f). These result resemble those found by Jaager and co-workers [41] (Tables 4 and 5), who suggested that the switch of stromal lineage to adipocyte-specific cell type require fewer genes than the switch to osteoblast- and chondrocyte-specific cell types. This apparently less specific MSC could sustain a less functionally specialised MSC in the adipose environment or possibly a more dormant cellular state.

Going further in the tissue-specificity analysis, CD and cytokine patterns of stromal cells were also studied. CD106 (vascular cell adhesion molecule VCAM1) appeared overexpressed exclusively in BM-MSCs. It has been long known that this marker is associated to bone marrow stromal cells. Moreover, a population of human cells with overexpression of two genes, VCAM1+/STRO1++, have been shown to develop bone tissue in vivo following ectopic transplantation to mice [43]. VCAM1 becomes down-regulated by cleavage during G-CSF mobilisation of HSPCs, conferring it an implied function in maintenance of the hematopoietic niches [44]. Other transcriptomic reports have also listed CD106 as expressed in BM-MSCs [31]. All these data pinpoint this gene as a BM-MSC specific gene. Beyond the bone marrow, other CD markers have been discovered for placental MSCs; like for example CD56 (NCAM1) that has been previously linked to highly clonogenic MSCs (i.e. small stromal cells that divide more rapidly and are detected frequently in preparations from younger donors) [45].

Another singular result from the tissue specificity analysis is the higher expression of TWIST1 and TWIST2 in dermal fibroblasts. The same outcome was previously associated to steady osteo-progenitor states of MSCs [43, 46]. TWIST1 and TWIST2 have been implicated in cell lineage determination and differentiation, therefore they may be up-regulated in more differentiated and committed stromal cells, as the fibroblasts are [46]. Their strong presence in skin fibroblasts compared to other stromal types involves a role in fate determination and more probably in the restriction of other differentiation paths. In fact, low expression of these genes has been correlated with low osteogenic differentiation potential.

Cytokines clustering around each tissue also defines the microenvironmental cues of specific signalisation. We found LEPR gene is specifically expressed in our human BM-MSCs. LEPR is the receptor for leptin, an adipocyte hormone that regulates body weight through fat metabolism, and has a role in hematopoietic pathways. In mice, sinusoid-associated leptin receptor (LEPR)^+^ cells maintain HSPC perivascular residence in the bone marrow [23]. In this way, LEPR+ stromal cells are key regulators of marrow homeostasis and the main bone producers in the adult mouse [47]. As far as we know, the expression of LEPR in humans had not been explicitly reported before.

CCR2 (CD192) activation by CCL2, as other receptor and cytokine partners found between HSPCs and BM-MSCs, mobilise monocytes during inflammatory responses and also seem to promote mobilisation of mesenchymal cells out of the marrow [48]. Activin receptors ACVR1 and ACVR2 also appear to be expressed in MSCs in the bone marrow. Type I and II receptors form a stable complex after ligand binding and transduce signals of BMPs and other TGFbeta family members. A mutation in ACVR1 (ALK2) is causative of fibrodysplasia ossificans progressiva, a disease that progresses with heterotopic ossifications in muscles, tendons, ligaments and general connective tissues. The mutation confers constitutive activity to the BMP type I receptor and sensitises mesenchymal cells to BMP-induced osteoblast differentiation and bone formation [49]. As a new finding, the production of the inhibin ligand by HSPCs (INHBC) may be controlling the balance of differentiation of MSCs toward osteoblasts. Functional experiments are needed to unravel the interplay effects of this pathway.

## Conclusions

Many molecular studies have shed light into the origin, identity and function of stromal cell populations within the bone marrow, although most of the knowledge has been only assayed on mouse models. The bulk of human MSCs currently in use for clinical therapies present a phenotype only partly described to date. Through the data provided in this work and the analytic methods developed, we were able to obtain a detailed profile of the transcriptional phenotype of human mesenchymal stem/stromal cells. Relationships based on transcriptomic distances were explored among different tissue-stromal cells and with other non-mesenchymal related cell types. To extract the common traits of the inherently heterogenic population of cultured MSCs from the bone marrow we applied a re-sampling protocol over a large integrated compendium of genome-wide expression data samples. We also extended the view to the interconnecting wires with hematopoietic housemates, performing a detailed comparison of the genome-wide expression profiles of MSCs and HSPCs. Up-regulated genes in the mesenchymal lineage against the hematopoietic lineage yielded a signature of 489 genes. Functional relationships were decrypted and potential regulatory genes of lineage commitment were postulated. Genes proposed for markers of stromal phenotype have been revised, including those that are CD markers, cytokines or regulatory elements like transcription factors. Tissue-specific gene expression sets associated to some stromal cell subtypes (i.e. AD-, BM- or PL-MSCs) were also uncovered. Finally, all the gene signatures and profiles depicted in this work are provided open to new investigations that may expand the understanding of the mesenchymal cell biology.

## Methods

### Isolation of human primary cells

All of the procedures performed in the current study with human samples are in accordance with the Declaration of Helsinki and collected after signed informed consent was obtained (as formally approved by the Ethics Committee of the Health District and the University Hospital of Salamanca).

Human AD-, BM- and PL- MSCs from healthy independent donors were isolated and expanded in vitro. Placental samples correspond to healthy newborns (*n* = 8), taken postpartum, immediately after delivering. Chorionic sections of 80 to 100 g were collected in aseptic conditions [50]. Each sample was washed thoroughly in normal saline solution, dissected into pea-sized fragments and enzymatically digested in 250 ml DMEM-LG medium (Gibco, Invitrogen), with 100 U/ml Collagenase type I (Gibco, Invitrogen) and 5 μg/ml DNase I (sterile, Roche). The mixture was incubated in a shaker for 2 h, at 37 °C [51, 52]. Cell suspensions were filtered through 70 μm strainers (Becton Dickinson), centrifuged (300xg, 10 min, 20 °C), resuspended in Hanks Solution (Gibco, Invitrogen) and processed for mononuclear fraction separation (MNCs). The bone marrow and adipose tissue samples correspond to healthy adult donors. Bone marrow samples of 10 to 20 ml from iliac crest aspirates (*n* = 5) were taken under local anaesthesia under institutional standards [53]. MNCs were separated by density gradient centrifugation using Ficoll-Paque® (GE Healthcare Bio-Sciences), then seeded on a plastic surface (10^6^ MNCs/cm^2^) with DMEM-LG supplemented with 10% FCS (BioWhittaker, Lonza) and 1% penicillin/streptomycin (Gibco, Invitrogen) [54]. Samples of adipose tissue lipoaspirates (*n* = 3) were harvested as described in [55]. Briefly, collected tissue was centrifuged (600 × g, 10 min, 20 °C), the cellular supernatant was separated and enzimatically digested (with 0.2% collagenase-I at 37 °C for 30 min). Tissue debris was filtered out with 70 μm strainers. Erythrocytes were lysed with ACK 1X lysing Buffer (A10492 Gibco, Invitrogen). The resulting cell suspensions were centrifuged (600 X g, 10 min, 20 °C), washed in PBS and plated under the same conditions as bone marrow MNCs.

Primary cultures of human skin fibroblasts (*n* = 5) from healthy adult donors were provided by the Tissue Engineering Unit, Community Centre for Blood and Tissues of Asturias [56], and INNOPROT (Ref: P10858: Human Dermal Fibroblasts, adult). Cells were grown in vitro for 2–3 passages under the same conditions as MSCs.

Leukapheresis samples (*n* = 3) from healthy adult donors were harnessed to isolate the fraction of mobilised hematopoietic progenitor cells (as in [57]). Immunomagnetic positive selection of CD34 cells was performed using the CD34 MicroBead Kit (MACS, Miltenyi Biotec) [58]. Isolated cell suspensions were then submitted to transcriptional analysis.

Cells were allowed to adhere for 3–5 days in a 37 °C, 5% CO_2_ atmosphere. The medium was completely changed twice a week thereafter. When confluence was reached, adherent cells were trypsinized (Trypsin-EDTA, Gibco, Invitrogen) and replated for culture expansion (seeding at 3,000-5,000 cells per cm^2^) [59]. Cell counts were performed within each passage.

Growth rates were evaluated calculating the population doubling times from the first to the sixth passage, following the formula: PDT = TExpan * (log(2) / log(Q2 /Q1)). The Wilcoxon test searched for significant differences between cells.

### Flow Cytometric controls

All stromal cells included in this study were tested by flow cytometry under the terms of the ISCT minimal criteria [20]. MSCs (~10^6^ cells) were harvested, resuspended in PBS, and incubated with conjugated antibodies using the following panel: CD90-FITC, CD14-PE, CD45-PerCP/CD34-FITP, CD73-PE, HLA-DR-PerCP/CD44-FITC, 166-PE, CD19-PerCP, CD105-APC/CD11b-FITC, CD33PE, 7AAD-PerCP (FITC: fluorescein isothiocyanate, PE: phycoerythrin, PerCP: peridinin chlorophyll protein, APC: allophycocyanin; Becton Dickinson Biosciences). 100,000 cell events per culture were acquired in a FACSCalibur flow cytometer (BD Biosciences) connected to the CellQuest program (BD Biosciences). Fluorescence-based expression of CD markers per event was analysed using the Infinicyt software (Cytognos).

### In vitro differentiation assays of MSCs

MSCs were plated and grown with each specific differentiation media (Miltenyi Biotec). For osteogenic and adipogenic induction, MSCs were adhered to 9.6 cm2 slide flasks (Nunc, Roskilde). Osteoblastic alkaline phosphatase activity was evaluated by NBT/BCIP colorimetric reactions (nitroblue tetrazolium chloride/5-bromo-4-chloro-3-indolyl-phophate) (Roche). Adipogenesis was observed by Oil-Red-O staining of lipid vacuoles (Certistain Merck KGaA). Pelleted cells placed in conical tubes were also conditioned towards chondrogenic differentiation. The resulting cells were embedded in paraffin, cut into 5 mm sections and Hematoxylin-Eosin-stained for evaluation of cartilage matrix formation [60].

### General calculations and statistics

All data analyses and graphics have been produced using the R statistic environment. General functions such as *boxplot*, *image, qplot (*from *ggplot2 library)* or *wilcox.test* were applied over the different data presented here. Specific methodologies are explicitly cited along different sections of this manuscript.

### Microarray data repository for a meta-analysis approach

A collection of 264 arrays were recruited from the GEO database [61]. Details and references of each dataset included are be found in Additional file 4. Data were mined considering the following inclusion criteria: (i) Technical issues: raw CEL files available, MIAME criteria surpassed, *Affymetrix* platform HG-U133 A and B (when both were available for each sample) or HG-U133 Plus 2.0. (ii) Biological issues: cell type (MSCs, HPCs/HSCs, other stromal cells), primary cell populations, outgo cytometric standards, non-pathological state, non-drug treated. (iii) Annotation issues (for research interest): tissue origin, culture passage, differentiation state, and differentiation method. Following these criteria, the whole compendium of samples collected for the study was 264, but this set was only used in the global clustering analysis, because for the differential expression comparisons we subselect only primary stem cells isolated from bone marrow (i.e. BM-MSCs and HSPCs from bone marrow), that are the ones included in the subset of 119 samples. In the set of 264 samples, as indicated in Additional file 4, there are quite a lot of MSC samples that correspond to stimulated cells (e.g. 24 samples from dataset GSE10315 were stimulated with BMP2 or with TGFB3) or differentiated cells (e.g. nine samples from dataset GSE9451 were MSCs differentiated to adipocytes, chondrocytes or osteoblasts). Moreover, in the compendium of 264 samples, some HSPCs were not isolated from bone marrow but from umbilical cord blood (e.g. eight samples from dataset GSE10438 and 9 samples from dataset GSE3823) or from whole peripheral blood (e.g. six samples from dataset GSE3823).

### Microarrays pre-processing and normalization

Different array platforms were all integrated in one. To place the whole dataset into the same analysis workflow, paired microarrays from HGU133A and HGU133B were unified into a new HGU133 Plus 2.0-based array. Union of the probes from both platforms were considered, and the highest intensity signal was taken into account when measures from the two platforms were available. Probe position coordinates were allocated following the Plus 2.0 scheme. An ad-hoc R chip definition file (CDFs) with complete unambiguous mapping of the probes from HGU133 microarrays to human genes (Emsembl) was used: *GeneMapper* R package published in [62]. In this way, the expression signal per gene was calculated. A compendium of 16,979 features uniquely identified as *Human Ensembl Genes* (ENSG IDs, Ensembl database 57) were computed per array. File reading and mapping was supported by the *ReadAffy* function from *affy library* in R-BioConductor.

Batch effect normalization was addressed by the *frozenRMA* method [63]. Batch-specific effects per probe were pre-computed using balanced subsets of the different batches recruited and then “frozen” into vectors. RMA normalization combining information contained in the frozen vectors was subsequently applied over the whole repository of arrays.

A sample-to-sample *Pearson* correlation heatmap of 264 arrays was produced using the *average* method of clustering (function *hclust*). Principal component analyses were conducted through distinct approaches: using genes or samples as variables. The *prcomp* function extracted the principal components that were graphically represented using the *biplot* and *scatterplot3d* tools, for two or three components, respectively. The *biplot* function generates a vector for each array in the recreated two-component-space.

### Resampling proceedings and differential expression (DE)

For DE analysis a resampling protocol was implemented in R, together with the *limma* package protocol to model and test the statistical contrasts. The set of 119 microarrays corresponding to 50 samples of BM-MSCs, 10 HSPCs, 11 FIBs, 13 OSTBs, 12 dOSTs and 23 stOSTs (Additional file 4) was used for differential expression analyses in 6 binary comparisons. These comparisons are detailed in Additional file 6, that shows four main contrasts designed. Contrasts 1, 2 and 3, compared BM-MSCs against other well-defined cell types: (1) BM-MSCs versus bone marrow HSPCs; (2) BM-MSCs versus skin fibroblasts (FIBs); and (3) BM-MSCs versus osteoblasts (OSTBs). A last group of contrasts (4A, 4B, 4C) compared several states derived from MSCs toward the osteoblastic lineage. These last comparisons were merely used for assaying the behavior of the resampling algorithm, as the data from these transformed MSCs were quite variable indicating that the experimental procedures applied produced strong and unpredictable effects in the cells. In the comparisons we labelled the groups as “Cell Type I” or “Cell Type II” (as indicated in columns B and C of the table in Additional file 6). The resampling approach took subsets of 7 by 7 samples with replacement (up to 200 times, i.e. 200 iterations), to produce a collection of DE results for each comparison. To be precise, each contrast designed with *limma* was re-sampled until the contrast results reached a plateau in which the genes being differentially expressed became constant. DE was considered statistically significant when the adjusted *p*-value was < 0.05 (adjusting for multiple-testing with FDR method). The stability behaviour of differentially expressed genes turned to be specific to each contrast. Thus, a different limit to the number of iterations was applied to each contrast (stability curves reached when the whole re-sampling workflow was run 100 times may be seen in Additional file 7). Once a threshold of iterations per contrast was fixed, genes presenting differential expression along all the re-sampling iterations were extracted and ranked. Then, the median of ranks was computed along all the iterations per DE gene. Each gene rank was assigned based on FDR adjusted *p*-values. DE genes were submitted to functional enrichment analysis through the DAVID web tool [64].

### *In-house* expression data set of tissue-MSCs, fibroblasts and HSPCs

Samples from AD-, BM- and PL- MSCs, skin fibroblasts and HSPCs were analysed using *Affymetrix* Human Exon 1.0 arrays. Cell samples were processed under the same controlled conditions. 15 microarrays were hybridised, including three biological replicates of each cell type. The platform reached higher coverage than the meta-analysis approach, attaining the measure of 20,238 unique human gene loci. The full expression signal of the arrays was normalised and calculated with *RMA algorithm* (*affy* package, R-Bioconductor [65]). The dataset is fully accessible from the GEO database under the identifier **GSE72332**.

Differential expression was tested using the *limma package* for the meta-analysis approach. Principal component analysis and heatmaps were done applying the same methods as in the meta-analysis approach. Likewise, the biomarker clustering heatmap was generated with the 358 CD marker genes retrieved from *Uniprot* and recognized as *Ensembl* genes.

### Cytokinome pattern analysis

The human pathway *Cytokine-cytokine receptor interaction* (*hsa:04060*) from KEGG database, containing 265 cytokine genes among the microarray genes, was used for the cytokinome mappings. The KEGG mapping tool was utilized. For the clustering analysis of cytokine profiles, we applied a filtering step of 50% to the less variable cytokines along stromal cell samples, and then a heatmap image of Euclidean distances (*average* clustering method) was produced.

### Mesenchymal signature, cross-validations, and functional enrichment analysis

Intersections of output gene lists from each comparative analysis were performed using the Ensembl gene symbols. To give strength to these output gene lists, our previously published RNA sequencing footprint of MSCs [16] was crossed with all the signatures produced in this study. Biological annotation and clustering of enrichment analysis were performed with the *Gene Term Linker* tool [66]. A network presenting the relationships between genes based on their shared enriched functions was generated using the *FGNet* package in R-Bioconductor [67]. Conservation scores from alignments of 100 vertebrate species (known as *UCSC hg19 phastCons*) were annotated with the *phastCons100way.UCSC.hg19* package*,* and then added to the functional network in yellow-blue colour scale.

## Additional files

Additional file 1: Recordings of established stromal cultures (AD-, PL-, BM- MSCs, and FIBs). Cell counts through passages (“Q1” and “Q2”), expansion times (“Texpan”) and doubling times (“DoubT”). (XLSX 52 kb)
Additional file 2: Flow cytometry histograms showing standard immunophenotyping of stromal cell populations studied. (PPTX 204 kb)
Additional file 3: Microscope images of the MSCs differentiation assays. In vitro multipotency detection by tri-lineage differentiation assays performed with samples of BM- PL- and AD- MSCs. Left-side photos show MSCs upon differentiation induction (positive assays). Negative controls (no induction medium applied) are shown in the photos on the right. (A) Osteogenic differentiation detected by alkaline phosphatase (AP) activity. Arrows indicate pools of high AP activity inside the cells. (B) Adipogenic differentiation detected by fat staining with Oil-Red-O. Arrows indicate fat vacuoles stained in red inside the cell cytoplasms. (C) Chondrogenic differentiation detected by tissue three-dimensional growth. Images show the section of cartilage spheroids stained with Hematoxilin-Eosin. Arrows denote areas of matrix composition produced by cells embedded in it. (PPTX 71553 kb)
Additional file 4: GEO dataset references of microarray data repository and number of samples selected in each of the two meta-analysis steps: (i) the hierarchical clustering analysis with 264 microarray samples (SET 264); and (ii) the differential expression resampling protocol with 119 microarray samples (SET 119). (XLSX 57 kb)
Additional file 5: Unsupervised hierarchical clustering of whole-genome expression profiles. The heatmap gives a comparative view of relationships among MSCs from three different tissues (bone marrow BM, placenta PL and adipose tissue AD), hematopoietic progenitor cells (HPC) and differentiated fibroblasts (FIB). All genes were used for the distance calculations. The dendrogram of the sample clustering is also shown. The colour scale provides a view of the distance range. (PPTX 164 kb)
Additional file 6: Output summary of the meta-analysis differential expression. Notations: BM-MSC = Bone Marrow MSC; HPC = Hematopoietic progenitor cells; FIB = fibroblasts; OSTB = Osteoblasts; dOST = MSC in-vitro derived osteoblasts; stOST = stimulated osteoblasts. The ”*n*” refers to the number of microarrays. (XLSX 39 kb)
Additional file 7: Performance curves of the re-sampling process that show the stability of the meta-analysis differential expression. Individual curves represent each contrast in the study, showing the number of differentially expressed genes accumulated (y-axis) across the iterations of the re-sampling protocol (x-axis). Each contrast with re-sampling has been run 100 times; the mean is shown with a red line. (PPTX 870 kb)
Additional file 8: KEGGmap “*hsa04060: Cytokine-cytokine receptor interaction*”. Red-colour boxes are differentially over-expressed genes in HPCs with respect to MSCs. Yellow coloured genes are over-expressed in MSCs relative to HPCs. (PNG 63 kb)
Additional file 9: List of 489 protein coding genes included in the mesenchymal lineage signature produced in this work. The table provides the gene symbols, ENSEMBL identifiers (IDs), corresponding UniProt IDs, protein names and description, as well as UniProt protein families and keywords. The proteins that are known CD markers (CDs) or transcription factors (TFs) are also indicated. (XLSX 129 kb)
Additional file 10: Table specifying the metagroups (“MG”) obtained from the functional enrichment analysis and subsequent annotation clustering of the mesenchymal lineage signature. The parameters used are detailed along the Materials and Results sections of the manuscript. Representative genes or terms of each metragroup are denoted in bold letters. (XLSX 38 kb)
Additional file 11: PCA of the 15 Exon-Arrays dataset using the 489 gene signature build for MSCs. (A) *Biplot* of the PCA outcome taking 489 genes as observations and 15 exon arrays as variables. (B) 3D plot illustrating the outcome of PCA taking the genes as variables and showing the 15 samples in the multidimensional space. (PPTX 140 kb)
Additional file 12: Conservation scores calculated with the *phastCons* alignment algorithm for 100 vertebrate species over the *UCSC hg19* genome. Only exonic regions were used in the calculation. (XLSX 30 kb)
Additional file 13: Gene coexpression network derived from the expression values of the 489 genes along the exon arrays in 15 samples of the different cell types studied. The coexpression was calculated using a *Pearson* correlation with a stringent cutoff to select the pairs with correlation coefficient *r* ≥ 0.95, allowing the selection of the best gene pairs. In this analysis the genes that correspond to known CD markers (CDs) and to transcription factors (TFs) are highlighted to illustrate possible interesting links. (PPTX 1551 kb)
Additional file 14: Cytoscape file (produced with Cytoscape version 3.1.0) that includes a gene coexpression network derived from the expression values of the 489 genes along the exon arrays in 15 samples of the different cell types studied. The coexpression was calculated using a *Pearson* correlation with a stringent cutoff to select the pairs with correlation coefficient *r* ≥ 0.95. The file includes only the gene pairs that passed the cutoff threshold, incorporating 447 genes and 9969 interactions. Several views of the network are included and various clusters of genes were produced as subnetworks. Biological information and annotation for individual genes is also provided. (CYS 781 kb)
Additional file 15: Functional enrichment analysis of the genes corresponding to the sets that are found overexpressed in the contrasts of each tissue-MSC subtype versus other mesenchymal/stromal subtypes. These gene lists are: 281 genes for AD-MSCs, and 412 genes for BM-MSCs and 531 genes for PL-MSCs. The file includes three tables with the data obtained with the DAVID functional enrichment tool. Each table includes the most relevant functional terms with significant *p*-values. (XLSX 68 kb)

## Abbreviations

AD: Adipose tissue
BM: Bone marrow
CD: Cluster of differentiation
cDNA: Complementary DNA
CFU-F: Colony-forming-unit of fibroblastic cells
DE: Differential expression
dOSTB: MSC-derived osteoblasts
ECM: Extracellular matrix
EMT: Epithelial to mesenchymal transition
FDR: False discovery rate
FIB: Fibroblast
hMSCs: Human mesenchymal stromal/stem cells
HPCs: Haematopoietic progenitor cells
HSCs: Haematopoietic stem cells
HSPCs: Haematopoietic stem and progenitor cells (CD34+ population)
ISCT: International society for cell therapy
MNCs: Mononuclear cells
MSCs: Mesenchymal stromal/stem cells
OSTB: Osteoblast
PCA: Principal component analysis
PL: Placenta
RNA-seq: Massively parallel sequencing of RNA technique
stOTB: Stimulated osteoblasts
TF: Transcription factor
TFBS: Transcription factor binding site
