# Supplementary material for: Insights into the human mesenchymal stromal/stem cell identity through integrative transcriptomic profiling
Source: BMC Genomics. 2016 Nov 21;17:944. doi: 10.1186/s12864-016-3230-0 (PMC5117530; doi:10.1186/s12864-016-3230-0)
Supplement: Additional file 2: — Flow cytometry histograms showing standard immunophenotyping of stromal cell populations studied. (PPTX 204 kb) [file 12864_2016_3230_MOESM2_ESM.pptx]

## Slide 1
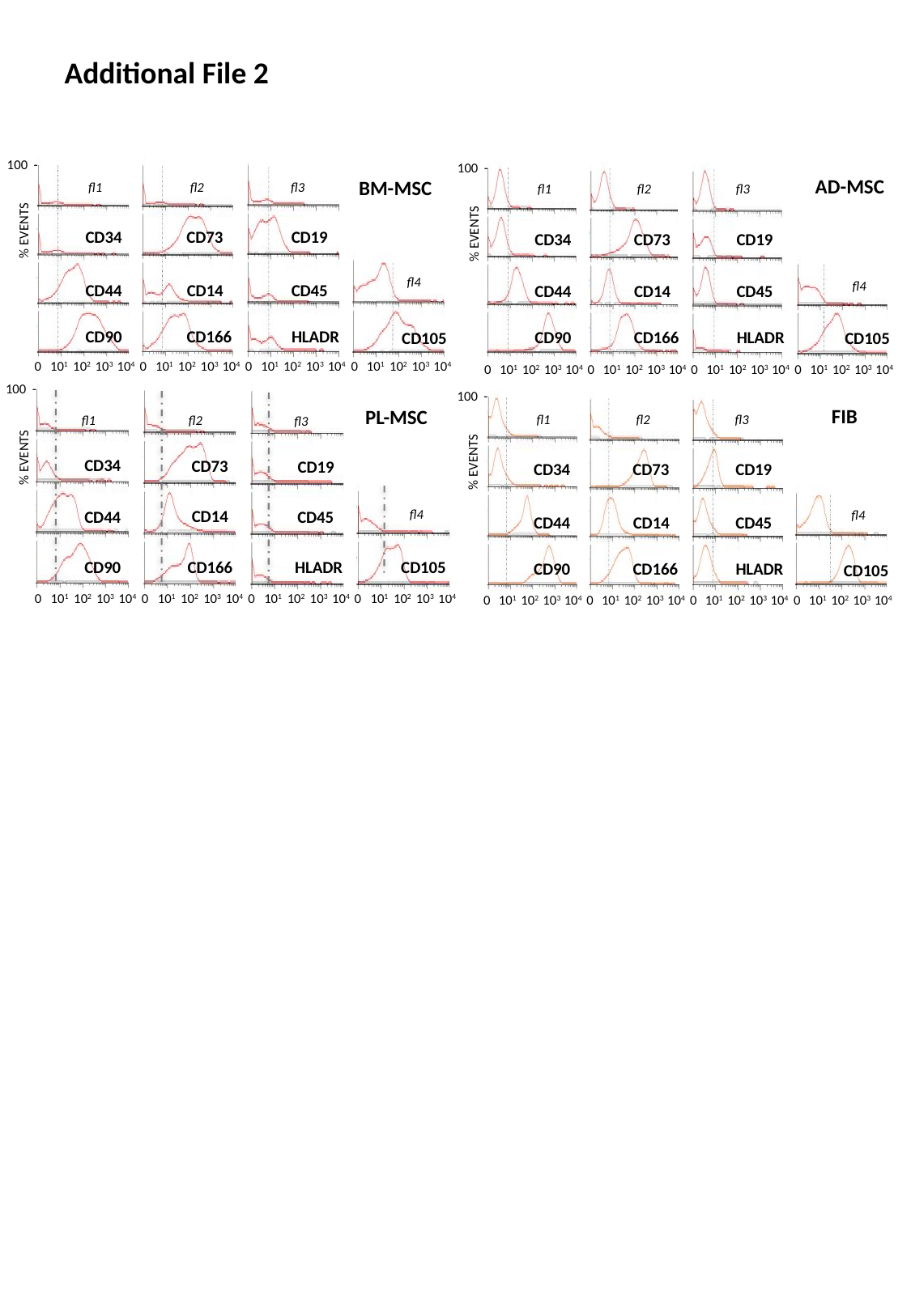

Additional File 2
100 -
BM-MSC
fl3
fl1
fl2
% EVENTS
CD34
CD73
CD19
fl4
CD14
CD45
CD44
CD90
CD166
HLADR
CD105
0
101
102
103
104
0
101
102
103
104
0
101
102
103
104
0
101
102
103
104
100 -
AD-MSC
fl3
fl1
fl2
% EVENTS
CD34
CD73
CD19
fl4
CD44
CD14
CD45
CD90
CD166
HLADR
CD105
0
101
102
103
104
0
101
102
103
104
0
101
102
103
104
0
101
102
103
104
100 -
PL-MSC
fl1
fl2
fl3
% EVENTS
CD34
CD73
CD19
fl4
CD14
CD44
CD45
CD90
CD166
HLADR
CD105
0
101
102
103
104
0
101
102
103
104
0
101
102
103
104
0
101
102
103
104
100 -
FIB
fl3
fl1
fl2
% EVENTS
CD34
CD73
CD19
fl4
CD44
CD14
CD45
CD90
CD166
HLADR
CD105
0
101
102
103
104
0
101
102
103
104
0
101
102
103
104
0
101
102
103
104
