# Supplementary material for: Insights into the human mesenchymal stromal/stem cell identity through integrative transcriptomic profiling
Source: BMC Genomics. 2016 Nov 21;17:944. doi: 10.1186/s12864-016-3230-0 (PMC5117530; doi:10.1186/s12864-016-3230-0)
Supplement: Additional file 3: — Microscope images of the MSCs differentiation assays. In vitro multipotency detection by tri-lineage differentiation assays performed with samples of BM- PL- and AD- MSCs. Left-side photos show MSCs upon differentiation induction (positive assays). Negative controls (no induction medium applied) are shown in the photos on the right. (A) Osteogenic differentiation detected by alkaline phosphatase (AP) activity. Arrows indicate pools of high AP activity inside the cells. (B) Adipogenic differentiation detected by fat staining with Oil-Red-O. Arrows indicate fat vacuoles stained in red inside the cell cytoplasms. (C) Chondrogenic differentiation detected by tissue three-dimensional growth. Images show the section of cartilage spheroids stained with Hematoxilin-Eosin. Arrows denote areas of matrix composition produced by cells embedded in it. (PPTX 71553 kb) [file 12864_2016_3230_MOESM3_ESM.pptx]

## Slide 1
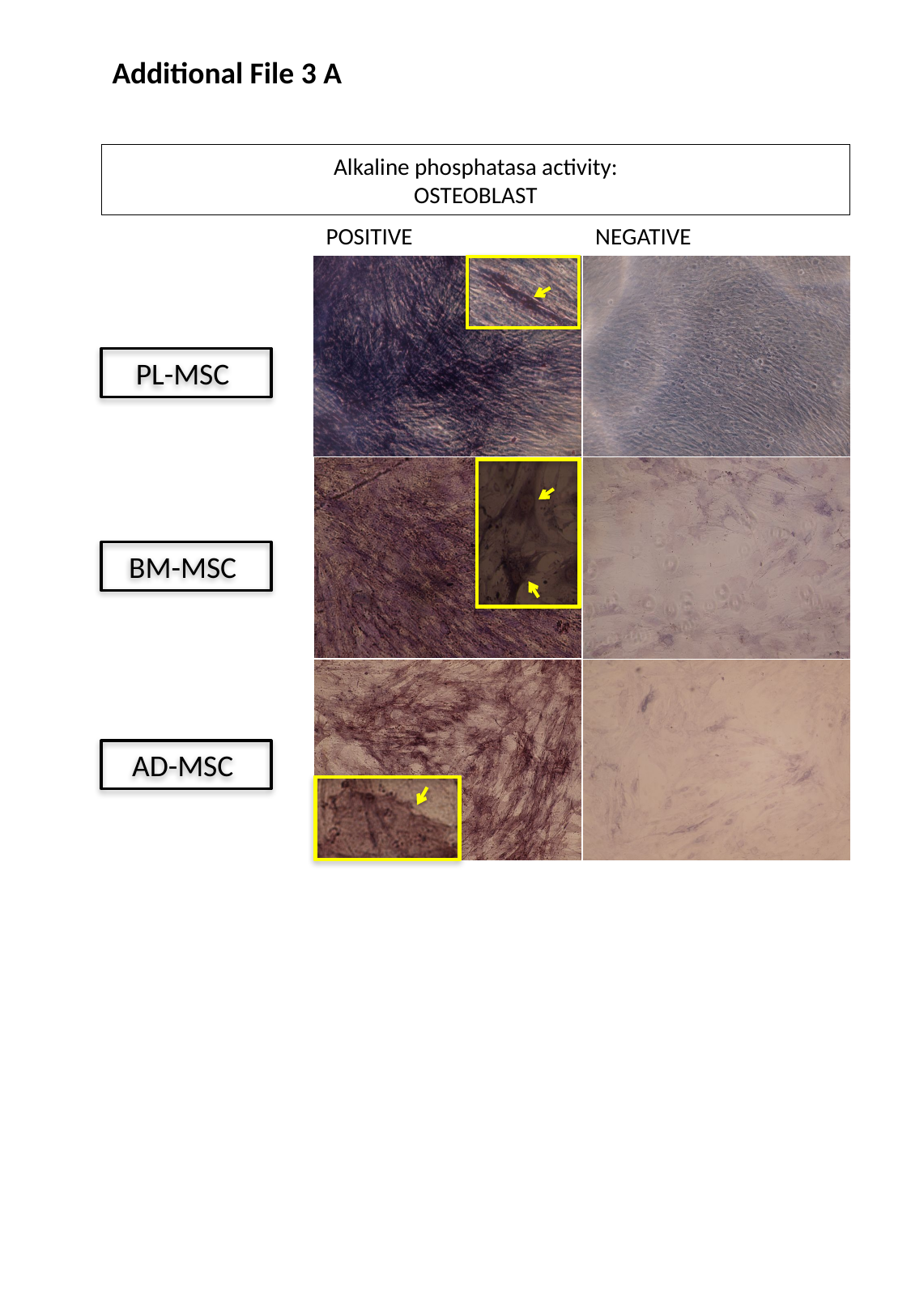

Additional File 3 A
# Alkaline phosphatasa activity: OSTEOBLAST
POSITIVE
NEGATIVE
PL-MSC
BM-MSC
AD-MSC

## Slide 2
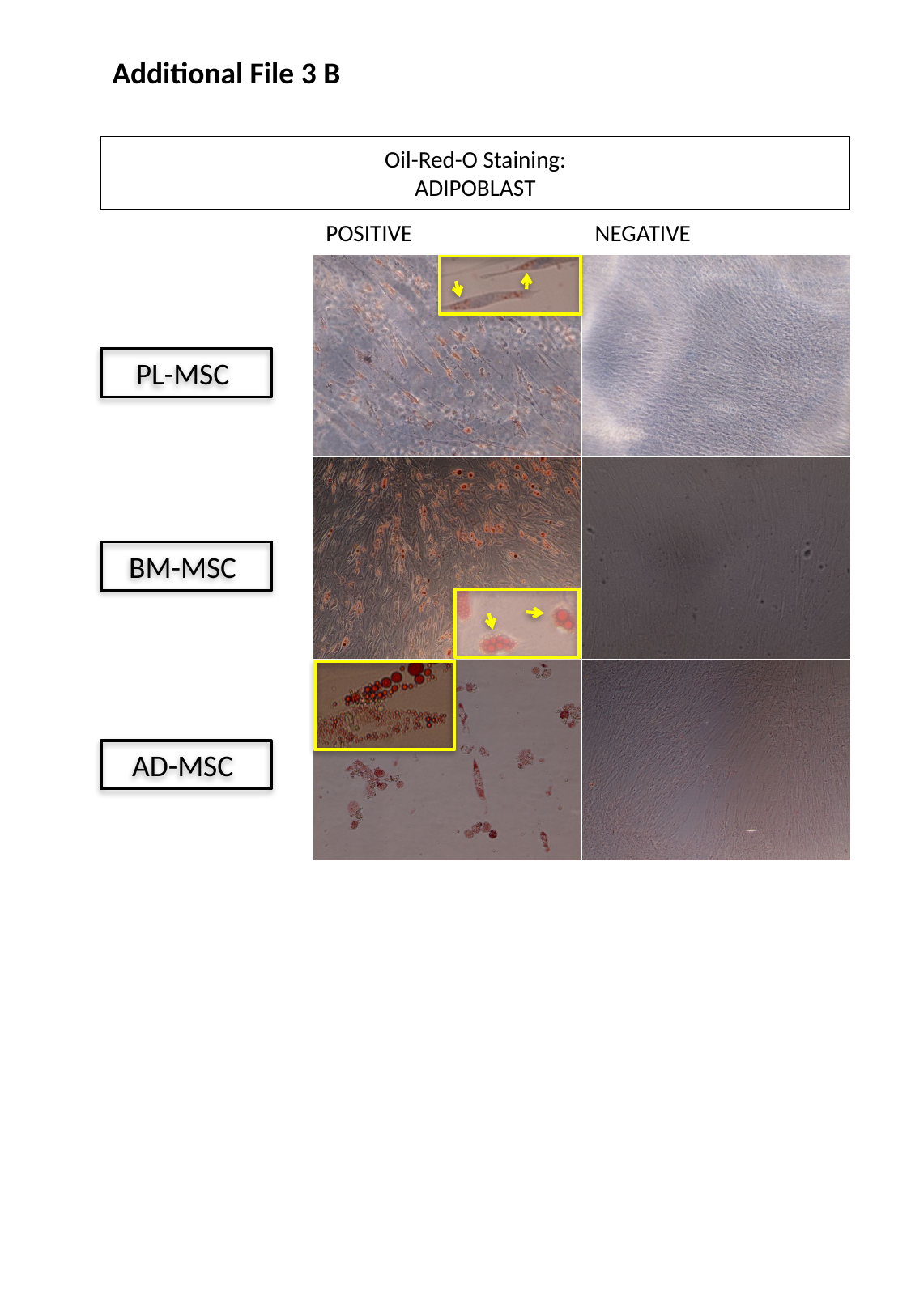

Additional File 3 B
Oil-Red-O Staining:
ADIPOBLAST
POSITIVE
NEGATIVE
PL-MSC
BM-MSC
AD-MSC

## Slide 3
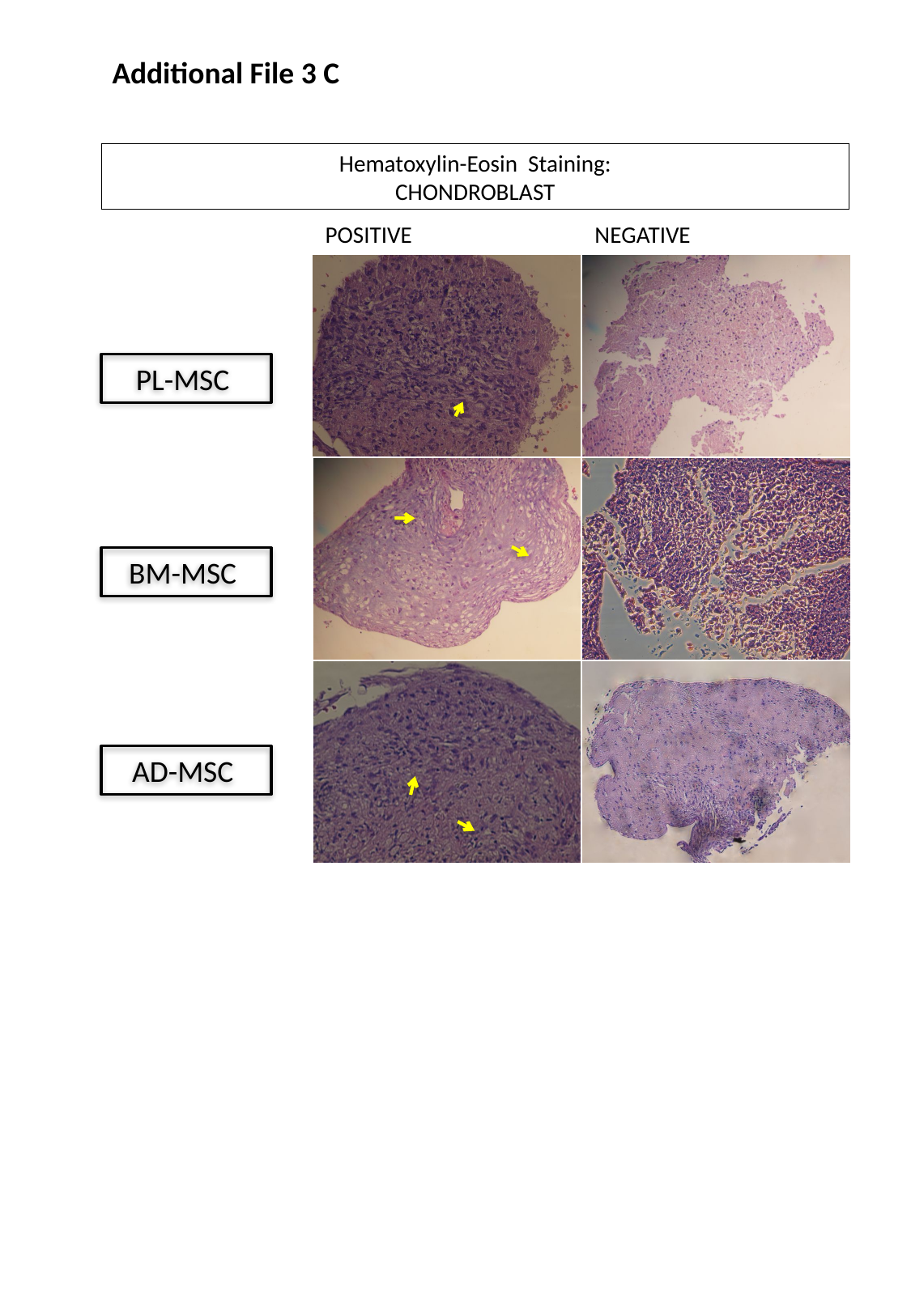

Additional File 3 C
# Hematoxylin-Eosin Staining: CHONDROBLAST
POSITIVE
NEGATIVE
PL-MSC
BM-MSC
AD-MSC
