# Supplementary figures and images for: Insights into the human mesenchymal stromal/stem cell identity through integrative transcriptomic profiling
Source: BMC Genomics. 2016 Nov 21;17:944. doi: 10.1186/s12864-016-3230-0 (PMC5117530; doi:10.1186/s12864-016-3230-0)

## Slide 1
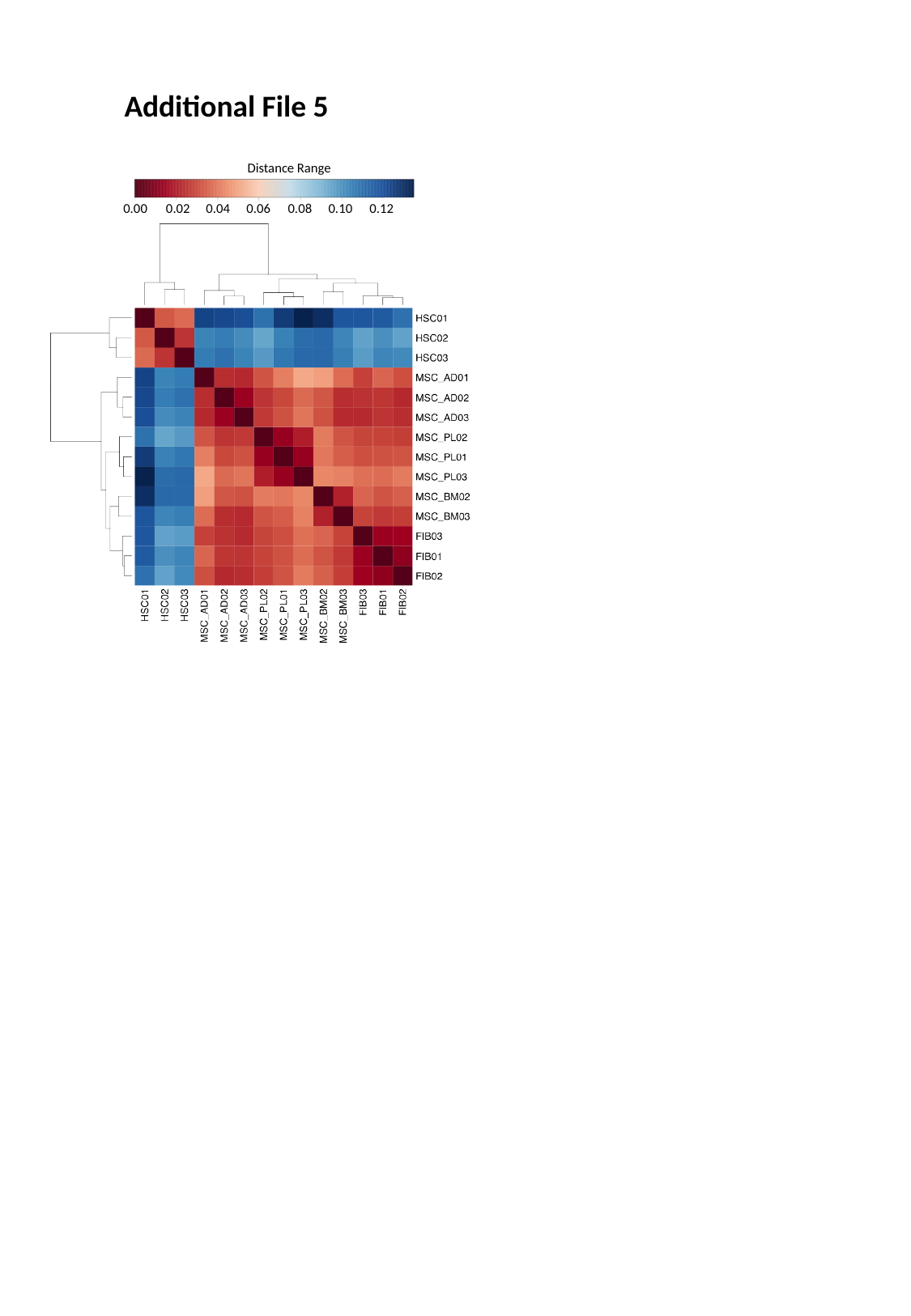

Additional File 5
Distance Range
0.02
0.04
0.06
0.12
0.00
0.08
0.10

Supplement: Additional file 5: — Unsupervised hierarchical clustering of whole-genome expression profiles. The heatmap gives a comparative view of relationships among MSCs from three different tissues (bone marrow BM, placenta PL and adipose tissue AD), hematopoietic progenitor cells (HPC) and differentiated fibroblasts (FIB). All genes were used for the distance calculations. The dendrogram of the sample clustering is also shown. The colour scale provides a view of the distance range. (PPTX 164 kb) [file 12864_2016_3230_MOESM5_ESM.pptx]

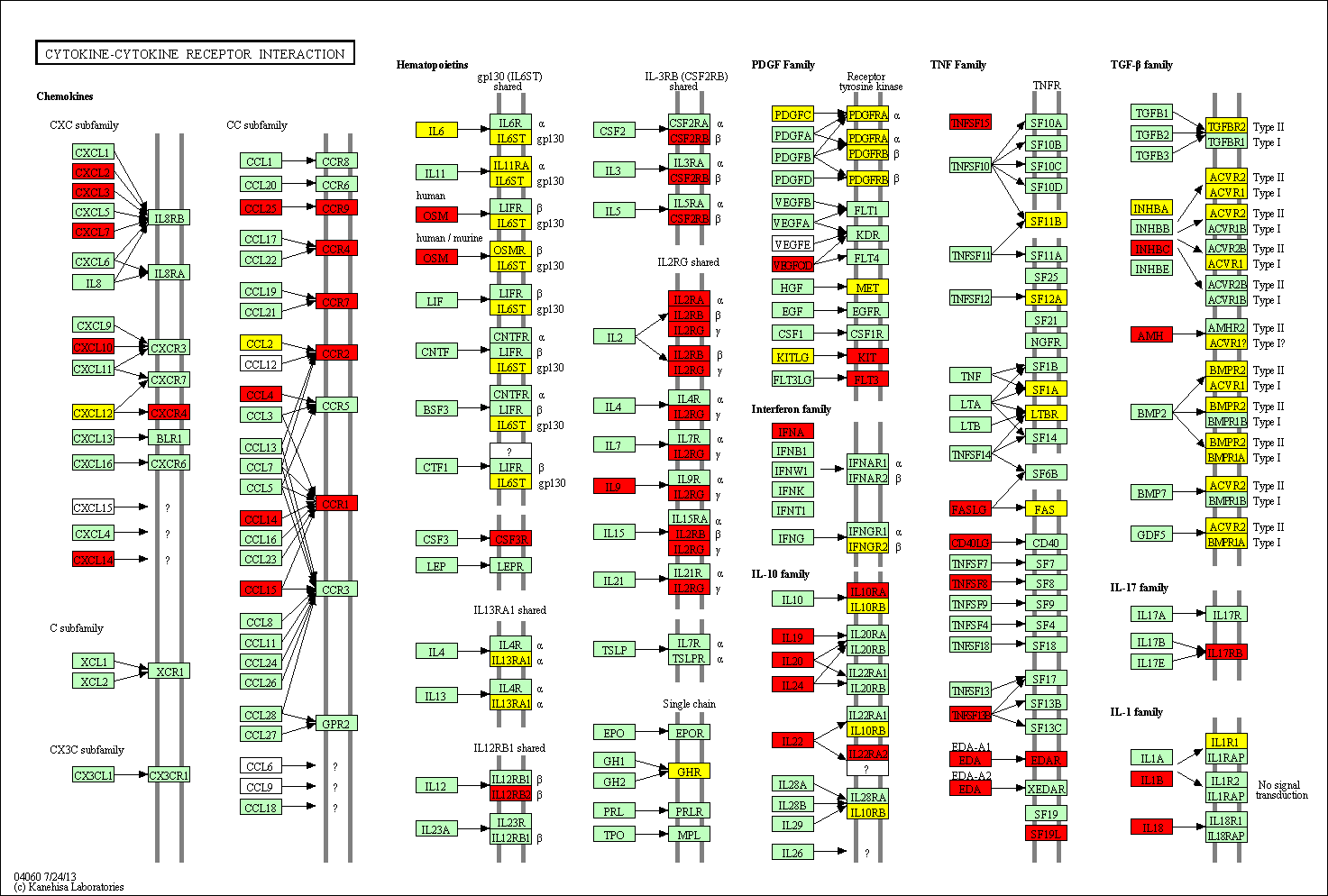

Supplement: Additional file 8: — KEGGmap “hsa04060: Cytokine-cytokine receptor interaction”. Red-colour boxes are differentially over-expressed genes in HPCs with respect to MSCs. Yellow coloured genes are over-expressed in MSCs relative to HPCs. (PNG 63 kb) [file 12864_2016_3230_MOESM8_ESM.png]

## Slide 1
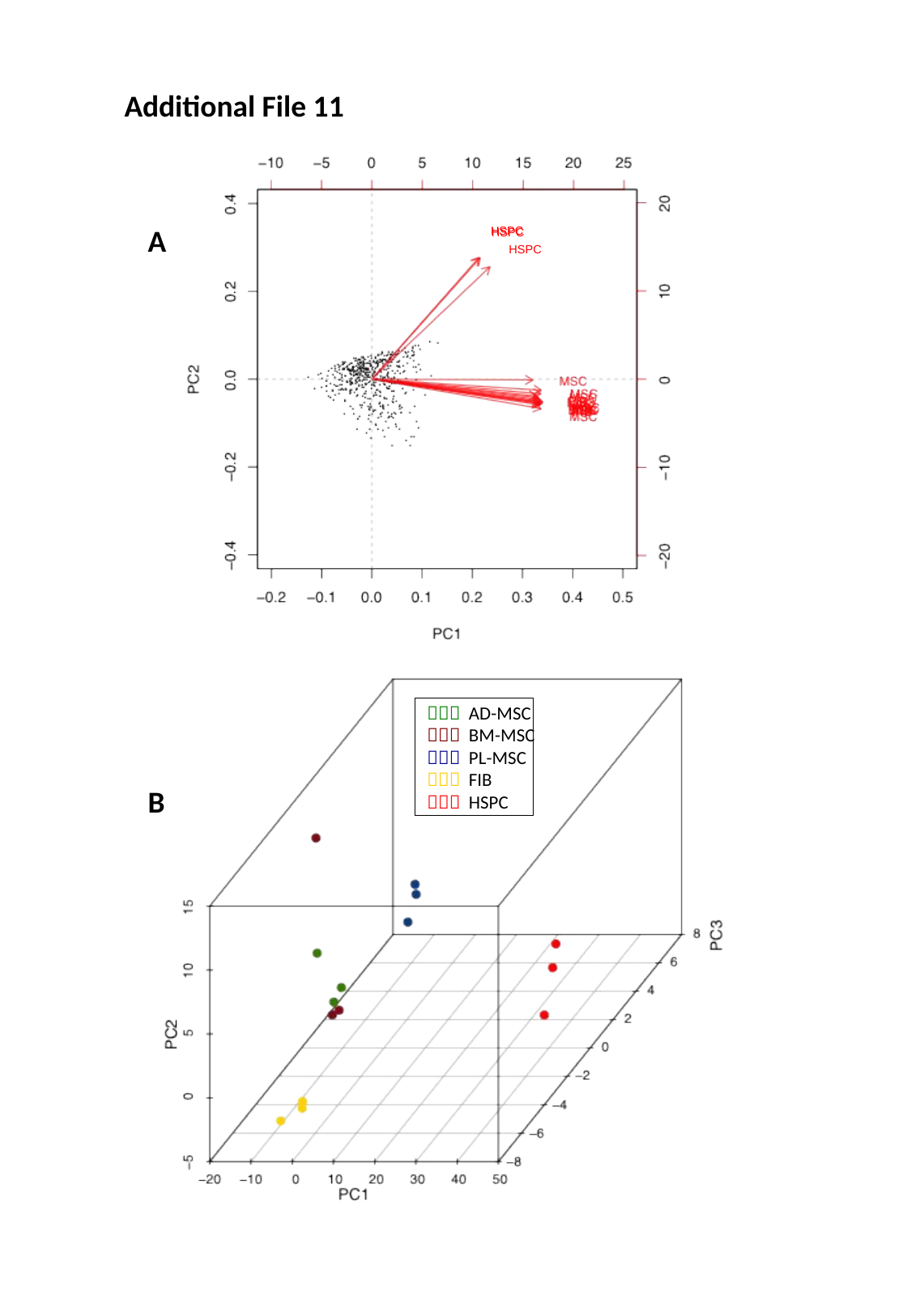

Additional File 11
A
HSPC
HSPC
HSPC
 AD-MSC
 BM-MSC
 PL-MSC
 FIB
 HSPC
B

Supplement: Additional file 11: — PCA of the 15 Exon-Arrays dataset using the 489 gene signature build for MSCs. (A) Biplot of the PCA outcome taking 489 genes as observations and 15 exon arrays as variables. (B) 3D plot illustrating the outcome of PCA taking the genes as variables and showing the 15 samples in the multidimensional space. (PPTX 140 kb) [file 12864_2016_3230_MOESM11_ESM.pptx]
