# Supplementary material for: Insights into the human mesenchymal stromal/stem cell identity through integrative transcriptomic profiling
Source: BMC Genomics. 2016 Nov 21;17:944. doi: 10.1186/s12864-016-3230-0 (PMC5117530; doi:10.1186/s12864-016-3230-0)
Supplement: Additional file 7: — Performance curves of the re-sampling process that show the stability of the meta-analysis differential expression. Individual curves represent each contrast in the study, showing the number of differentially expressed genes accumulated (y-axis) across the iterations of the re-sampling protocol (x-axis). Each contrast with re-sampling has been run 100 times; the mean is shown with a red line. (PPTX 870 kb) [file 12864_2016_3230_MOESM7_ESM.pptx]

## Slide 1
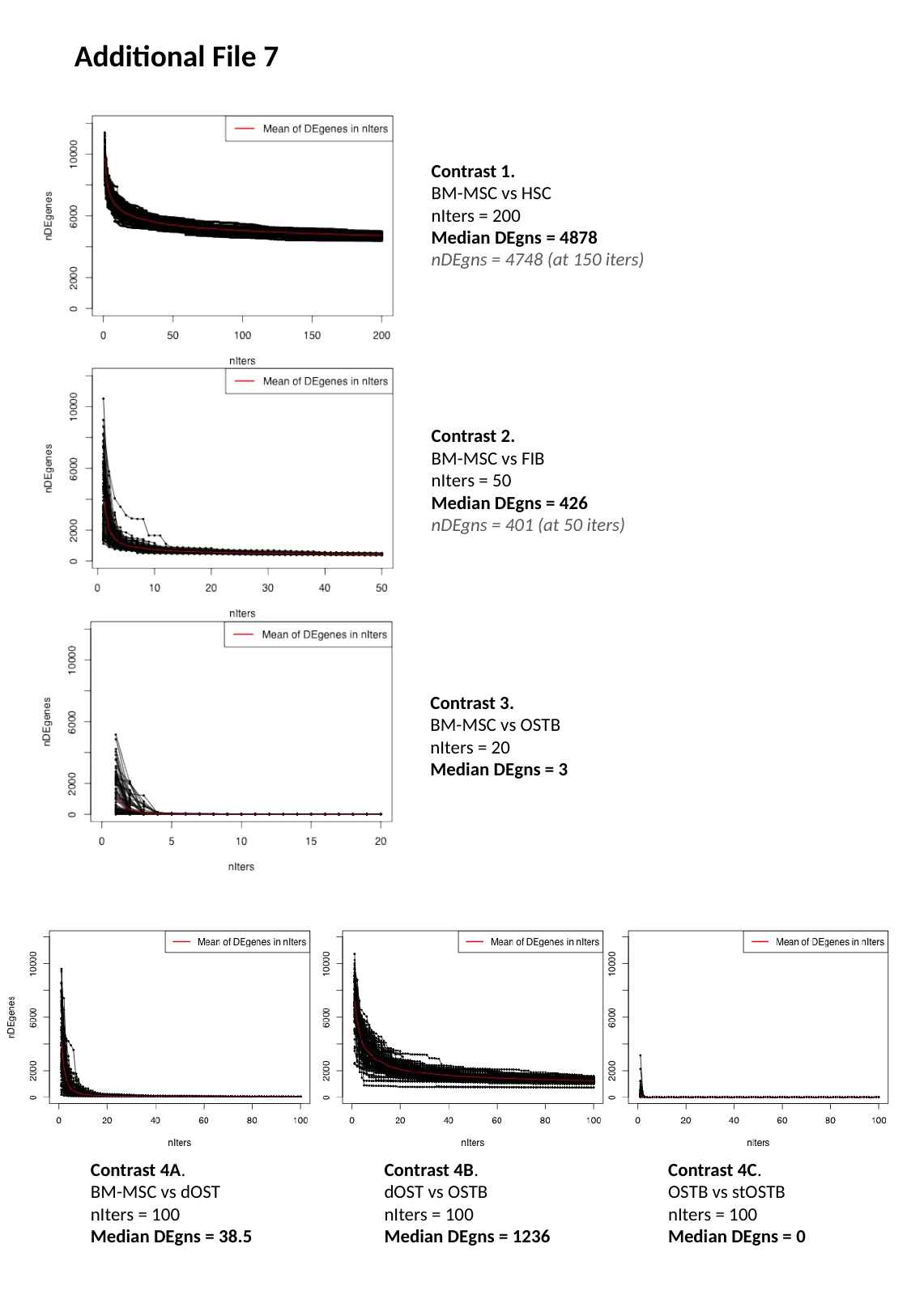

Additional File 7
Contrast 1.
BM-MSC vs HSC
nIters = 200
Median DEgns = 4878
nDEgns = 4748 (at 150 iters)
Contrast 2.
BM-MSC vs FIB
nIters = 50
Median DEgns = 426
nDEgns = 401 (at 50 iters)
Contrast 3.
BM-MSC vs OSTB
nIters = 20
Median DEgns = 3
Contrast 4A.
BM-MSC vs dOST
nIters = 100
Median DEgns = 38.5
Contrast 4B.
dOST vs OSTB
nIters = 100
Median DEgns = 1236
Contrast 4C.
OSTB vs stOSTB
nIters = 100
Median DEgns = 0
