# Supplementary material for: Insights into the human mesenchymal stromal/stem cell identity through integrative transcriptomic profiling
Source: BMC Genomics. 2016 Nov 21;17:944. doi: 10.1186/s12864-016-3230-0 (PMC5117530; doi:10.1186/s12864-016-3230-0)
Supplement: Additional file 13: — Gene coexpression network derived from the expression values of the 489 genes along the exon arrays in 15 samples of the different cell types studied. The coexpression was calculated using a Pearson correlation with a stringent cutoff to select the pairs with correlation coefficient r ≥ 0.95, allowing the selection of the best gene pairs. In this analysis the genes that correspond to known CD markers (CDs) and to transcription factors (TFs) are highlighted to illustrate possible interesting links. (PPTX 1551 kb) [file 12864_2016_3230_MOESM13_ESM.pptx]

## Slide 1
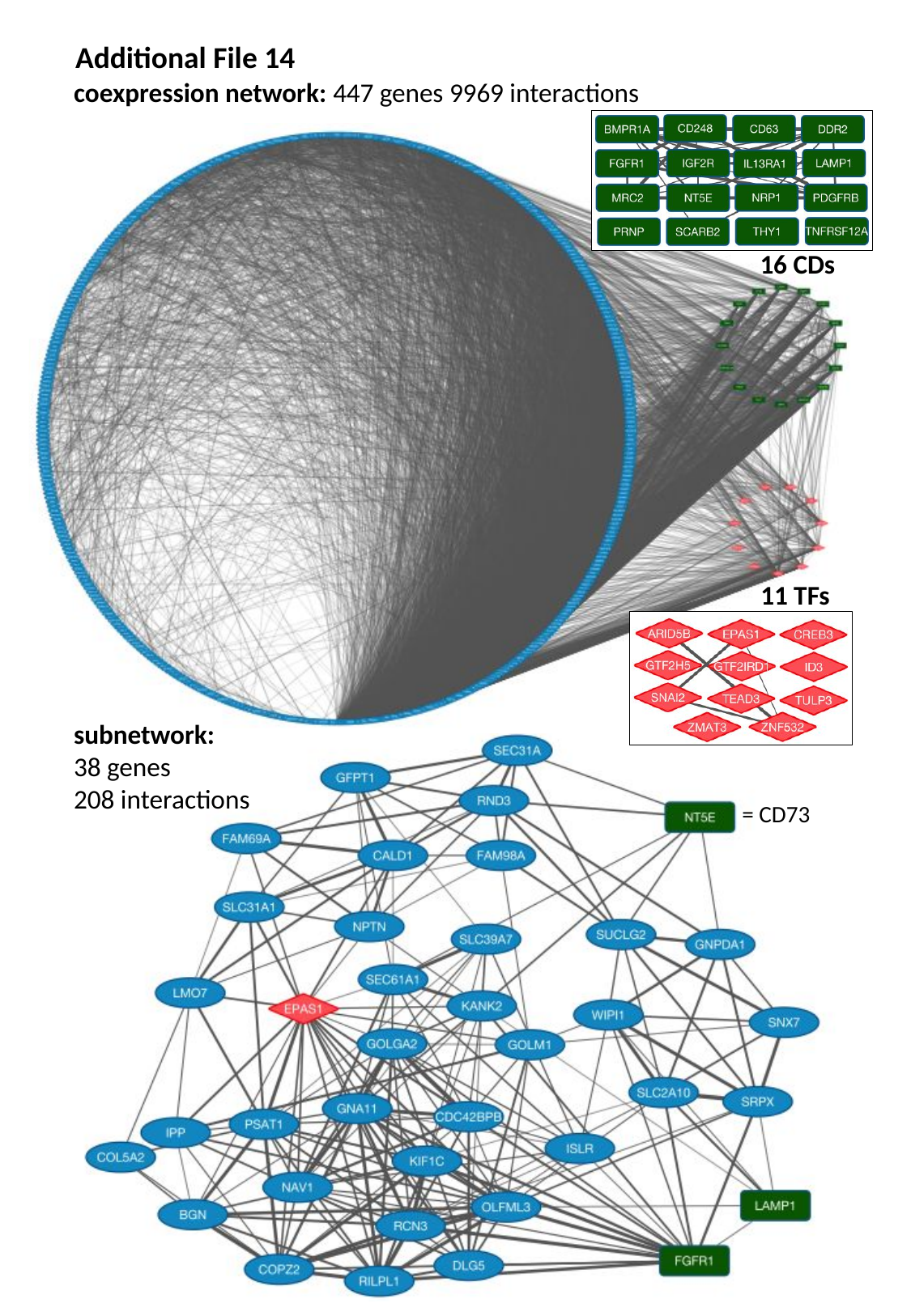

Additional File 14
coexpression network: 447 genes 9969 interactions
16 CDs
11 TFs
subnetwork:
38 genes
208 interactions
= CD73
